# Decreasing the RAG:SAG ratio of granola cereal predictably reduces postprandial glucose and insulin responses: a report of four randomised trials in healthy adults

**DOI:** 10.1017/jns.2022.22

**Published:** 2022-03-17

**Authors:** Thomas M.S. Wolever, Alexandra L. Jenkins, Janice E. Campbell, Adish Ezatagha, Simarata Dhillon, Jodee Johnson, John Schuette, Yumin Chen, YiFang Chu

**Affiliations:** 1 INQUIS Clinical Research, Toronto, Ontario, Canada; 2 PepsiCo, Inc., R&D Health & Nutrition Sciences, Barrington, IL, USA; 3 PepsiCo, Inc., R&D Measurement Sciences, Barrington, IL, USA

**Keywords:** Glycaemic response, Rapidly available glucose, Slowly available glucose, Starch, avCHO, available dietary carbohydrates, BMI, body mass index, CG, control granola, GI, glycaemic index, iAUC, incremental areas under the curve, RAG, rapidly available glucose, RGR, relative glycaemic responses, RHR, relative hunger responses, RIR, relative insulinaemic responses, SAG, slowly available glucose, TG, test granola

## Abstract

Dietary starch contains rapidly (RAG) and slowly available glucose (SAG). To establish the relationships between the RAG:SAG ratio and postprandial glucose, insulin and hunger, we measured postprandial responses elicited by test meals varying in the RAG:SAG ratio in *n* 160 healthy adults, each of whom participated in one of four randomised cross-over studies (*n* 40 each): a pilot trial comparing six chews (RAG:SAG ratio 2·4–42·7) and three studies comparing a test granola (TG1-3, RAG:SAG ratio 4·5–5·2) with a control granola (CG1–3, RAG:SAG ratio 54·8–69·3). Within studies, test meals were matched for fat, protein and available carbohydrate. Blood glucose, serum insulin and subjective hunger were measured for 3 h. Data were subjected to repeated-measures analysis of variance (ANOVA). The relationships between the RAG:SAG ratio and postprandial end points were determined by regression analysis. In the pilot trial, 0–2 h glucose incremental areas under the curve (iAUC0–2; primary end point) varied across the six chews (*P* = 0·014) with each 50 % reduction in the RAG:SAG ratio reducing relative glucose response by 4·0 %. TGs1-3 elicited significantly lower glucose iAUC0–2 than CGs1–3 by 17, 18 and 17 %, respectively (similar to the 15 % reduction predicted by the pilot trial). The combined means ± sem (*n* 120) for TC and CG were glucose iAUC0–2, 98 ± 4 *v*. 118 ± 4 mmol × min/l (*P* < 0·001), and insulin iAUC0–2, 153 ± 9 *v*. 184 ± 11 nmol × h/l (*P* < 0·001), respectively. Neither postprandial hunger nor glucose or hunger increments 2 h after eating differed significantly between TG and CG. We concluded that TGs with RAG:SAG ratios <5·5 predictably reduced glycaemic and insulinaemic responses compared with CGs with RAG:SAG ratios >54. However, compared with CG, TG did not reduce postprandial hunger or delay the return of glucose or hunger to baseline.

## Introduction

Classifying available dietary carbohydrates (avCHO) by the degree of polymerisation (DP) into mono- and di-saccharides (sugars), oligosaccharides (polymers with DP, 3–9) and starch (glucose polymers with DP > 9) predicts neither acute physiological effects (such as glycaemic response)^(1)^ nor major health outcomes^(2–6)^. The physiological impact of avCHO depends, at least in part, on its rate of digestion in the small intestine and the type of monosaccharides absorbed^(7)^. Starch that is slowly digested *in vitro* elicits low glycaemic and insulinaemic responses *in vivo*^(8,9)^, effects that are generally considered to be beneficial^(10,11)^. The glycaemic impact of dietary starch is influenced by its chemical structure^(12)^, which depends on plant variety^(13,14)^ and its physico-chemical properties such as the degree of starch gelatinisation^(15,16)^, food matrix^(17)^ and particle size^(18,19)^, and may be altered by chemical or enzymatic treatment^(20)^, food processing^(21,22)^ and by the addition of fat and protein^(23–25)^.

Englyst *et al.* proposed that the glycemic impact of foods could be explained by their content of rapidly available glucose (RAG, the amount of glucose from rapidly digested starch and free sugars that is rapidly available for absorption) and slowly available glucose (SAG, the amount of glucose from slowly digested starch and free sugars that is slowly available for absorption)^(26,27)^. However, in almost all previous studies examining the effect of foods containing different amounts of RAG and SAG on glycaemic and insulinaemic responses, the amounts of fat^(9,27–31)^ and, in some cases, protein^(9,27)^ and avCHO^(30,31)^ contained in the test meals, varied by physiologically important amounts. Since fat, protein and avCHO have independent effects on glucose and insulin responses^(32–34)^, such differences confound the effects of RAG and SAG on postprandial responses. Thus, the magnitude of the independent effect of RAG and SAG on glucose and insulin responses is not clear. We aimed to address this question by assessing the effect of varying in RAG and SAG while controlling for fat, protein and avCHO.

We performed a pilot study using virtually protein-free, low-fat chews equivalent in avCHO but varying in RAG and SAG to determine the dose–response relationship between the RAG:SAG ratio and postprandial glucose, insulin and hunger responses. Then, studies were done to see if the effect on postprandial responses of altering the RAG:SAG ratio in granola cereals containing moderate amounts of protein and fat was equivalent to that in the chews.

We studied granola because it is a popular food, commonly consumed as a breakfast or snack, that may contain a variety of ingredients, including oats, wheat, puffed rice, nuts, seeds and dried fruits that contribute moderate amounts of protein and fat. The types of ingredients and how they are processed, as well as how the final granola product is processed, affect its RAG and SAG content. We developed three different test granolas (TGs) with low RAG:SAG ratios and compared their effects on glucose, insulin and hunger responses with three control granolas (CGs) matched with the TGs for fat, protein and avCHO content. Our objectives were to compare postprandial glucose, insulin and hunger responses elicited by each TG with those after its CG and to see if the magnitude of these effects was predicted by the results in the chews.

## Materials and methods

We conducted four randomised cross-over studies, each of which compared, in *n* 40 participants, the postprandial glucose, insulin and hunger responses elicited by test meals containing equivalent amounts of avCHO, but with different RAG:SAG ratios. All four studies were conducted at INQUIS Clinical Research (formerly GI Labs), a contract research organisation. The dates for the first and last screening visits and the first and last study visits, respectively, were as follows (dd/mm/yy): pilot study, 08/05/17 to 17/07/17 and 11/05/17 to 11/08/17; granola study 1, 15/05/17 to 07/07/17 and 17/05/17 to 24/07/17; granola study 2, 30/08/17 to 10/11/17 and 01/09/17 to 1711/17; and granola study 3, 19/01/18 to 27/04/18 and 24/01/18 to 09/05/18. The protocol for each of the four studies was the same except for different test meals and a different group of participants. The pilot study was not registered, but the three granola studies were registered at www.clinicaltrials.gov as NCT03495778, NCT03493022 and NCT03491514.

### Participants

Participants, who were allowed to participate in only one of the four studies, were males and non-pregnant females aged 18–65 years with body mass index (BMI) ≥21·0 and <32·0 kg/m² and fasting serum glucose <7·0 mmol/l (or whole blood glucose <6·3 mmol/l). They were recruited from the pool of volunteers who had previously participated in studies at INQUIS and had given permission to be contacted for future studies and from advertisements on popular websites. Detailed lists of inclusion/exclusion criteria are given in Supplementary Methods. Each of the four studies was conducted according to the guidelines laid down in the Declaration of Helsinki, and all procedures involving human subjects were approved by the Western Institutional Review Board (pilot trial, approved 16 April 2017, WIRB protocol no. 20170863; granola study 1, approved 5 May 2017, WIRB protocol no. 20170868; granola study 2, approved 24 August 2017, WIRB protocol no. 20171847; granola study 3, approved 11 January 2018, WIRB protocol no. 20173053). Written informed consent was obtained from all subjects.

### Study design and procedures

Each study had a randomised cross-over design. The pilot study was single-blinded (subjects blinded to treatments), while the three granola studies were double-blinded (subjects and outcome assessors blinded to treatments). Participants willing to be considered came to the research centre to have the study procedures explained to them and be given a copy of the consent form, which they could either sign then, take away to sign later or decline to participate. The participants were encouraged to ask any questions they may have had and not to sign the consent form until all their questions had been answered to their satisfaction. Those who consented to participate came to INQUIS for a pre-selection visit when eligibility was determined by asking questions about medical history and drug use, measuring height and weight and calculating BMI; if fasting glucose within the last 3 months was not available, arrangements were made to have it measured.

In the pilot study, eligible participants were studied on 6 separate days over a period of 11–37 d. Most of the intervals between individual test days were between 2–7 d (*n* 193, 96·5 %), with *n* 6 (3 %) being 8–14 d and *n* 1 being 18 d. In the three granola studies, the eligible participants were studied on 2 separate days over a period of 3–30 d. The interval between tests was 2–7 d for *n* 105 (88 %), 8–14 d for *n* 11 (9 %) and 15–29 d for *n* 4 (3 %). On each test day, the participants came to INQUIS in the morning after a 10–12 h overnight fast (water was allowed during this fasting period).

The procedures were the same for all four studies. The participants were asked to maintain stable dietary and activity habits throughout their participation and to refrain from drinking alcohol and from unusual levels of food intake or physical activity for 24 h before each test. If any subject was unwell or had not complied with the preceding instruction, the test was cancelled and rescheduled for another day.

On each test occasion, after being weighed and giving two fasting blood samples, 5 min apart, the subjects started to consume a test meal. The subjects were asked to consume the entire test meal within 10 min. At the first bite, a timer was started, and additional blood samples were taken at 15, 30, 45, 60, 75, 90, 105, 120, 150 and 180 min after they started eating. The blood samples were obtained by finger prick; two to three drops of blood were placed into a fluoro-oxalate tube for glucose analysis and six to eight drops into a 0·3 ml Microvette (Sarstedt Inc., Numbrecht, Germany) for insulin analysis. If a participant's hands were cool, the hands were warmed with an electric heating pad for 3–5 min prior to each sample. A glass of water (250 ml) was offered between 120 and 180 min.

After each blood sample had been obtained, subjective hunger was assessed by the visual analogue scale (VAS) using the following question from the Motivation to Eat questionnaire^(31)^: ‘How hungry do you feel?’. The VAS was a 100- mm horizontal line anchored at the left end with the statement ‘not hungry at all’ and at the right end with ‘as hungry as I have ever felt’; the subjects made a vertical mark along the line to indicate their feelings at that moment. After the last hunger rating had been completed, the participants were offered a snack and allowed to leave.

### Test meals

*Pilot study:* the test foods consisted of ready-to-eat chews. After production, the chews remained fresh for approximately 6 weeks. Since it was anticipated that it would take about 3 months to complete all subject visits, the chews were produced in three batches. Each batch was analyzed for RAG and SAG using the Englyst method^(27)^ (Supplementary Table S1). Each test meal was served with a drink of one or two cups of coffee or tea or water with 30 ml of 2 % milk and non-caloric sweetener if desired; at the first visit, each subject selected the type and volume of drink desired and the same type and volume was consumed at each subsequent visit.

*Granola studies*: The test foods consisted of granola cereals served with 122·5 g skim milk. Prior to each study, the CGs and TGs were analyzed for RAG and SAG using the Englyst method^(27)^. TG1 and TG2 formulas included oats, wheat, sugar, canola oil, whey, inulin, non-fat dry milk, molasses, whey protein concentrate, honey, flavour, sunflower oil, water, natural flavour, tocopherols and sulphites. The difference between TG1 and TG2 formulas was the type of oats used. CG1 and CG2 formulas included wheat, oat, sugar, canola oil, puffed rice, puffed millet, water, tapioca syrup, inulin, maltodextrin, whey protein concentrate, maple flavour, cane sugar flavour and sunflower oil. For TG3 and CG3, raisin and almond inclusions were added to the formulas. The portion sizes and nutrient composition of the test meals are shown in Table 1.

**Table 1.** Composition of test meals

Test meal	Abbreviation	Weight (g)	Energy (kcal)	Protein (g)	Fat (g)	Carbohydrate (g)				
						Avail	Fibre	RAG	SAG	RAG:SAG
Pilot study^a^										
Chew 1 c40-15	C1	61·2	160·2	0·1	1·6	35·0	12·9	23·7	10·0	2·4
Chew 2 c30-8	C2	81·3	194·7	0·1	2·1	35·0	29·4	24·9	7·9	3·2
Chew 3 c40-8	C3	66·9	176·1	0·1	1·7	35·0	17·6	27·6	6·0	4·6
Chew 4 c50-8	C4	57·0	143·6	0·1	1·4	35·0	9·2	28·5	5·2	5·5
Chew 5 c40-4	C5	78·6	172·2	0·1	2·0	35·0	27·2	31·6	2·4	13·4
Chew 6 c40-0·1	C6	77·4	180·8	0·1	1·9	35·0	26·8	31·4	0·7	42·7
Granola study										
Test granola #1	TG1	50·5	210	5·2	5·3	32·9	5·1	22·2	5·0	4·5
Control granola #1	CG1	54·3	210	4·9	5·2	32·9	4·7	32·7	0·60	54·8
Test granola #2	TG2	50·5	210	5·2	5·3	32·9	5·1	22·1	4·24	5·2
Control granola #2	CG2	54·3	210	4·9	5·2	32·9	4·7	32·3	0·47	69·3
Test granola #3	TG3	51·0	209	4·9	5·2	33·4	4·7	17·3	3·62	5·0
Control granola #3	CG3	54·1	209	4·6	5·2	33·3	4·4	29·1	0·50	58·6
Skim milk^b^	–	122·5	42	4·1	0·1	6·1	0·0	–	–	–

Avail, available carbohydrate (total carbohydrate minus dietary fibre rounded to the nearest 0·1 g); RAG, rapidly available glucose; SAG, slowly available glucose.

a RAG and SAG values calculated from the means of the three batches produced (Supplementary Table S1).

b Skim milk added to all test meals.

### Randomisation and concealment

*Pilot study*: a table containing forty-eight orders of the six treatments were created such that each treatment occurred (1st, 2nd, 3rd, 4th, 5th and 6th) eight times; they were randomly ordered using the RAND() function on an excel spreadsheet. Only the subjects were blinded to treatment by virtue of the fact that all six chews were similar in appearance and taste. *Granola studies:* for each study, a table of forty-eight orders of two treatments was created using the RAND() function in blocks of unequal sizes such that, in each block, the Control and Test treatments came first an equal number of times. Outcome assessors and subjects were blinded by virtue of the fact that two test meals were similar in appearance and taste and were provided by the sponsor in coded packages; the code was not broken until the database had been checked and locked. For all four studies, treatment sequences were created by the principal investigator and were assigned to subjects in the order of their appearance for the first visit by the clinical research coordinator. Participants replacing drop-outs were assigned the next unused sequence.

### Biochemical analysis

Tubes containing blood for glucose analysis were rotated to mix the blood with the anti-coagulant, stored at 4°C until the last blood sample had been collected and then at −20°C until analysis (performed within 3 d) using a YSI model 2300 STAT analyzer (Yellow Springs, OH). The microvette tubes containing blood for insulin were left at room temperature to allow the blood to clot, centrifuged and the serum transferred to labelled polypropylene tubes and stored at −20 °C prior to analysis using the Human Insulin EIA Kit (catalog # 80-INSHU-E10.1, Alpco Diagnostics, Salem, NH). The performance of the glucose and insulin analyses and how missing and undetectable values were handled are described in Supplementary Methods.

### Calculations

Incremental areas under the blood glucose and serum insulin response curves (iAUC), ignoring the area below fasting, were calculated using the trapezoid rule^(35,36)^ for the time intervals 0–2 h (iAUC0–2), 2–3 h (iAUC2–3) and 0–3 h (iAUC0–3). Since self-reported hunger decreased after eating, the total areas under the curve (tAUC; area below the curve down to a score of 0) were calculated for the hunger scores^(37)^. Fasting glucose, insulin and hunger were taken to be the mean of the values for the two fasting samples. Fasting insulin resistance (HOMA-IR) and β-cell function (HOMA-B) were calculated using the homeostasis model assessment^(38)^ and the mean of the four measures (−5 and 0 min for each of two test meals) of fasting glucose and insulin in each subject. Relative glycaemic responses (RGR), insulinaemic responses (RIR) and hunger responses (RHR) were calculated as follows: for the pilot study, each participant's iAUC0–2 (glucose and insulin) or tAUC0–2 (hunger) was expressed as a percentage of the mean for all six chews, and the values were then adjusted so that the mean for C6 =100 %; for the granola studies, each participant's iAUC0–2 or tAUC0–2 after TG was expressed as a percentage of that after CG. Because of high within-subject variation, the distributions of TG/CG values are skewed to the right, leading to falsely high means and SDs. To correct this, outlying values of RGR, RIR and RHR (defined as values >2 × sd above the mean) were excluded.

### Statistical analysis

The prespecified primary end point for each study was glucose iAUC0–2; the prespecified secondary end points included glucose iAUC from 2–3 and 0–3 h, glucose increment at 2 h, insulin iAUC from 0–2, 2–3 and 0–3 h, and hunger increment at 2 and 3 h. We also assessed hunger tAUC from 0–2, 2–3 and 0–3 h, RGR, RIR and RHR. With *n* 40 subjects, each study had 80 % power to detect a difference of 16 % in the primary end point of glucose iAUC0-2.

Pilot study data were analysed using repeated-measures analysis of variance (ANOVA) examining for the main effects of test meals. After a demonstration of significant heterogeneity, individual means were compared using Tukey's test to adjust for multiple comparisons. In each granola study, end points for TG were compared with those for CG by the paired *t*-test. For each of the four studies, the criterion for statistical significance was a two-tailed *P* < 0·05. The relationships between the RAG, SAG and log2(RAG:SAG ratio) of the test meals and means of the glucose, insulin and hunger responses were assessed using Pearson's correlation coefficients; the slopes and elevations of the regression lines for the pilot study data were compared with those for the granola study data using GraphPad Prism version 9.2.1 (GraphPad Software, San Diego, CA).

Since the results showed that the mean glucose iAUC0–2 for the three CGs (118, 118 and 119 mmol × min/l) and the three TGs (98, 96 and 99 mmol × min/l) were virtually identical to each other, in a *post-hoc* analysis, the data from the three granola studies were combined and analyzed by ANOVA examining for the main effects of test meals and study, and the study × test meal interaction. Using this database, we also determined the independent associations between test meals and subject characteristics (age, sex, ethnicity, height, weight, BMI, fasting glucose, fasting insulin and for insulin end points, the respective glucose end point) and the primary and secondary end points by multiple linear regression using the step-up method with dummy variables for sex (male *v*. female) and ethnicity (Caucasian *v*. non-Caucasian). Multiple regression analysis including all the variables was also performed.

## Results

### Participants

**Fig. 1.**
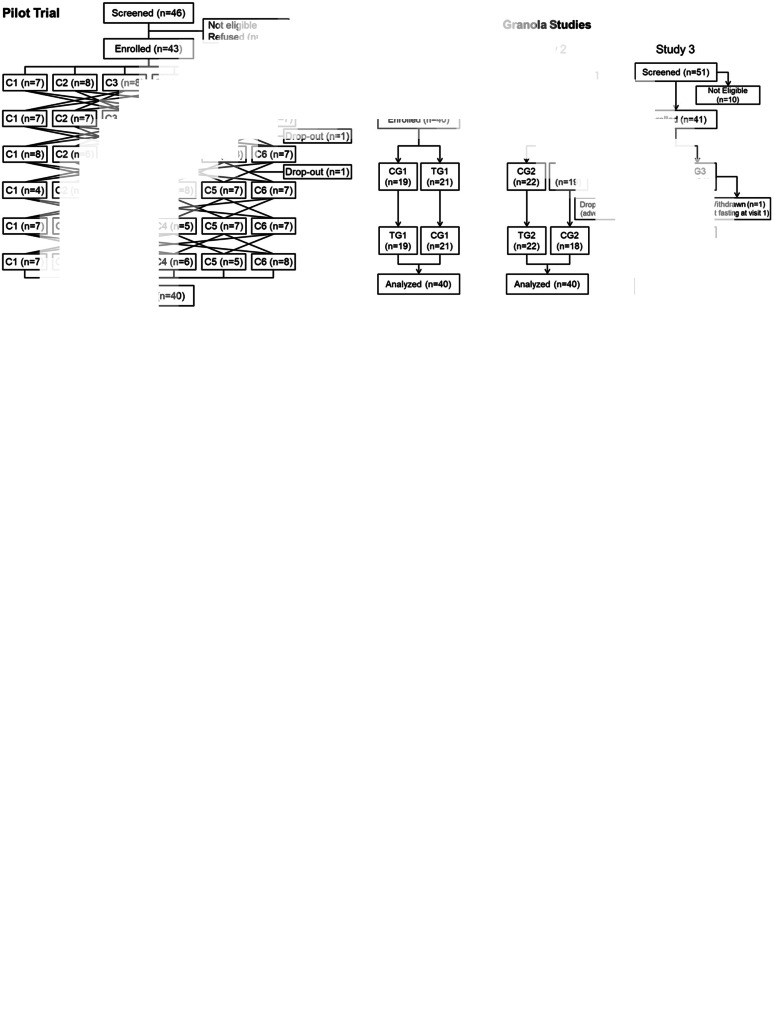
Flowcharts. The reasons for participants dropping out of the pilot trial were as follows: inability to chew the test meals that got stuck in the participant's dentures (*n* 1) and changing personal time commitments (*n* 2). In granola study 2, the participant who dropped out vomited after the fasting finger-stick blood sample and before consuming the test meal and did not wish to continue. In granola study 3, a participant was withdrawn because of a protocol violation (the participant was not fasting; see Supplementary Results for details).

*Pilot trial:* forty-six subjects were screened, of whom forty-three were enrolled and forty were completed the study (Fig. 1). The completers were twenty-five males and fifteen females (*n* 20 Caucasian) with a combined age of 39 ± 12 years (mean ± sd), body mass index 26·0 ± 3·1 kg/m² (twenty-two with BMI ≥25 kg/m²), blood pressure 116 ± 13/71 ± 10 mmHg and fasting blood glucose 4·36 ± 0·49 mmol/l (Table 2). Stable doses of supplements were taken by five subjects and stable doses of allowed medications by five (Supplementary Table S2). Minor protocol violations are described in Supplementary Results. No adverse events were reported.

**Table 2.** Participant characteristics

		Pilot (*n* 40)	Granola studies			
			Study 1 (*n* 40)	Study 2 (*n* 40)	Study 3 (*n* 40)	Total (*n* 120)
Male:female (% male)		25:15 (60)	23:17 (58)	26:14 (65)	18:22 (45)	67:53 (56)
Ethnicity	White	20 (50)	20 (50)	22 (55)	22 (55)	64 (53·3)
	Aboriginal	1 (2·5)	1 (2·5)	0	0	1 (0·8)
	Arab/W Asian	1 (2·5)	2 (5)	2 (5)	0	4 (3·3)
	Black	7 (17·5)	8 (20)	3 (7·5)	4 (10)	15 (12·5)
	Chinese	6 (15)	3 (7·5)	5 (12·5)	3 (7·5)	11 (9·2)
	Filipino	0	2 (5)	1 (2·5)	1 (2·5)	4 (3·3)
	Korean	2 (5)	0	0	0	0
	Latin American	1 (2·5)	1 (2·5)	3 (7·5)	4 (10)	8 (6·7)
	S Asian	1 (2·5)	3 (7·5)	3 (7·5)	5 (12·5)	11 (9·2)
	SE Asian	1 (2·5)	0	1 (2·5)	1 (2·5)	2 (1·7)
Age (year)		38·8 ± 12·0	37·1 ± 14·5	35·7 ± 13·8	32·7 ± 13·2	35·2 ± 13·9
Height (cm)		171 ± 10	172 ± 9^ab^	173 ± 8^a^	168 ± 10^b^	171 ± 9
Weight (kg)		76·3 ± 12·9	74·7 ± 12·4^ab^	75·7 ± 11·3^a^	68·3 ± 13·6^b^	72·9 ± 12·8
BMI (kg/m²)		26·0 ± 3·0*	25·3 ± 3·1	25·2 ± 2·6	24·0 ± 2·6	24·8 ± 2·8
BMI ≥ 25·0 (%)		22 (55 %)	17 (43 %)^ab^	22 (55 %)^a^	10 (25 %)^b^	49 (41 %)
Systolic BP (mmHg)		116 ± 12·6	116 ± 11·6	121 ± 13·3	116 ± 10·9	118 ± 12
Diastolic BP (mmHg)		71 ± 10	69 ± 8·9	72 ± 8·9	69 ± 6·9	70 ± 8·4
Fasting glucose (mmol/l)		4·48 ± 0·39	4·40 ± 0·33	4·43 ± 0·34	4·33 ± 0·32	4·39 ± 0·33
Fasting insulin (pmol/l)		25·5 ± 13·3	22·1 ± 7·8^b^	27·6 ± 23·0^b^	39·6 ± 18·1^a^	29·8 ± 18·9
HOMA-IR		0·86 ± 0·51	0·83 ± 0·29^b^	1·05 ± 0·91^b^	1·49 ± 0·71^a^	1·12 ± 0·73
HOMA-B		93·1 ± 40·8	48·3 ± 24·8^b^	58·9 ± 45·5^b^	87·3 ± 36·5^a^	64·8 ± 39·9
HOMA-IR ≥ 1 (%)		8 (20 %)	8 (20 %)^a^	9 (23 %)^a^	30 (75 %)^b^	47 (39 %)

Values are numbers (%) or means±sd for *n* 40 in each study.

BMI, body mass index; BP, blood pressure; HOMA-IR, homeostasis model assessment insulin resistance; HOMA-B, homeostasis model assessment β-cell function.

^ab^Numbers (%) or means with different letter superscripts differ significantly by the *χ*² test or Tukey's test, respectively (*P* < 0·05).

* Mean differs significantly from totals for granola studies by the two-tailed *t*-test (*P* < 0·05).

*Granola studies*: altogether 158 subjects were screened, of whom 123 were enrolled and 120 completed the studies (Fig. 1). Of the 120 who completed sixty-seven (56 %) were male, sixty-one (51 %) were Caucasian and forty-nine (41 %) were overweight or obese. Participants in the three studies were of similar age, sex, ethnicity, BMI, blood pressure and fasting glucose (Table 2). However, compared with the participants in study 2, the participants in study 3 were shorter, weighed less and fewer were overweight or obese. Nevertheless, study 3 participants had a higher fasting insulin, HOMA-IR and HOMA-B than those of studies 1 and 2 (Table 2). Stable doses of supplements were taken by ten subjects, stable doses of allowed medications by seventeen and stable doses of both supplements and medications by four (Supplementary Table S2).

Across all three trials, there were nine minor protocol violations and three non-serious adverse events (Supplementary Results). Details about the performance of the glucose and insulin analyses are given in Supplementary Methods.

### Pilot trial end points

**Fig. 2.**
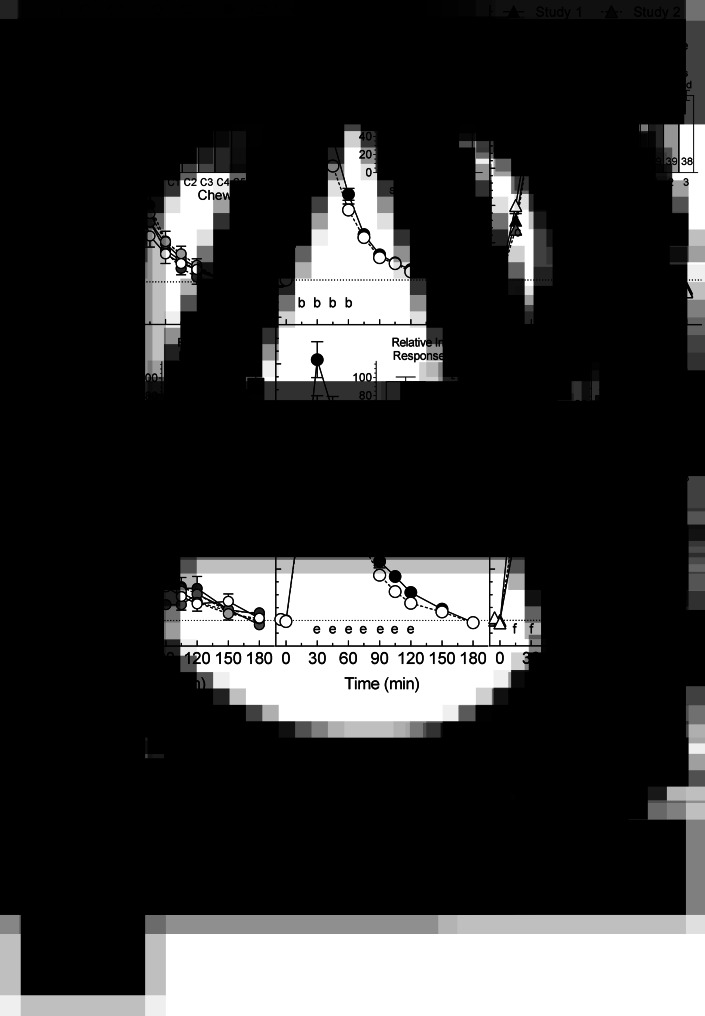
Glucose and insulin responses. Glucose (A–C) and insulin (D–F) increments elicited by: (A) and (D), six chews (C1–C6) (pilot trial, means ± sem, *n* 40); (B) and (E), three control and three test granolas (pooled means ± sem, *n* 120); (C) and (F), subjects in the three granola studies (pooled means ± sem, *n* 40). Insets (A) and (D) show iAUC0–2 for the six chews expressed as a percentage of C6 normalised, so the mean for C6 = 100 (means ± sem, *n* 40). Insets (B), (C), (D) and (E) show iAUC0–2 for test granola expressed as a percentage of control before and after excluding outliers >2 × sd from the mean (means ± sem and *n*). ^a^Significant differences between: C6vC1 at 30, 45, 60 and 150 min; and C6vC2, C6vC3 and C4vC1 at 45 min (Tukey's *P* < 0·05). ^b,e^Significant difference between control and test granola [analysis of variance (ANOVA), *P* < 0·05]. ^d^Significant differences between: C6vC1 and C6vC2 at 30, 45 and 60 min; C6vC3 at 45 and 60 min; C5vC2 at 30 min; and C4vC1 at 60 and 75 min (Tukey's *P* < 0·05). ^f^Significant differences between: studies 1 and 3 at 15 and 30 min; and studies 1 and 2 at 45 min (Tukey's *P* < 0·05). ^xy^Means with different letter superscripts differ significantly (Tukey's *P* < 0·05). ^z^Relative response significantly less than 100 (*t*-test, *P* < 0·05).

**Fig. 3.**
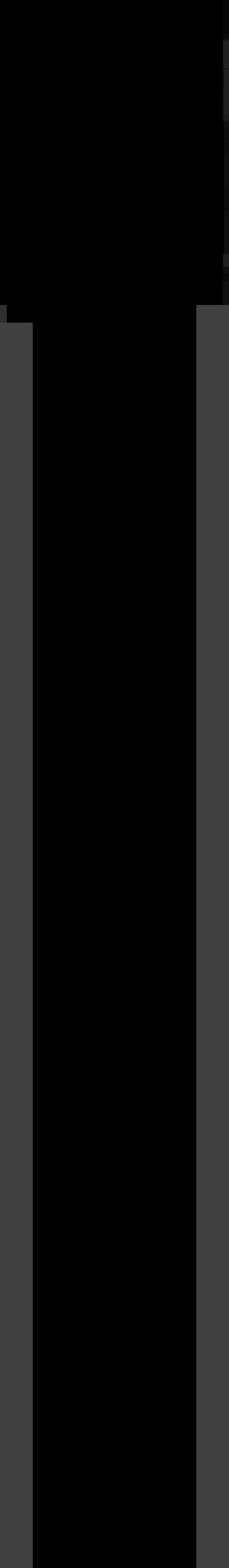
Associations between relative responses and rapidly available glucose (RAG), slowly available glucose (SAG) and log2(RAG:SAG). Values are means ± sem for *n* 40 subjects. The circles show the results for the pilot study; the black, grey and white triangles, respectively, show the results for granola studies 1, 2 and 3. Lines are regression lines for the pilot study (solid) and granola studies (dashed). Correlation coefficients (*r*) and *P*-values are given for the pilot and granola study data. The slopes and elevations of the regression lines for pilot *v*. granola do not differ significantly in panels (A), (C), (E), (H) and (I) (Supplementary Table S4). The slopes of the regression lines for pilot and granola data differ significantly in (B), (D) and (F) (*P* < 0·05). The elevation of the regression lines for pilot and granola data in (G) differs significantly (*P* < 0·05).

Fasting glucose was similar among chews (*P* = 0·68) varying from (mean ± sem) 4·45 ± 0·07 (C6) to 4·53 ± 0·08 mmol/l (C2). Mean glucose increments differed among chews at 30, 45, 60 and 150 min (Fig. 2(A)) but not at 2 h (Table 3). Glucose iAUC0-2 differed among chews (*F*_(5,195)_ 2·93, *P* = 0·01) with C1 being significantly less than C6 (Table 3). The RGR for C1, 78·2 ± 3·6 %, was significantly less than that for C6, 100 ± 4·5 % (Fig. 2(A)). Means for iAUC2-3 and iAUC0-3 did not differ significantly. Means for glucose iAUC0-2 and iAUC0-3, respectively, were positively related to RAG (*P* = 0.06 and *P* = 0·04) and log2(RAG:SAG) (*P* = 0.01 and *P*=0·009) and negatively related to SAG (*P* = 0.02 and *P* = 0·009; Supplementary Figs. S1(A)–(C) and S2(A)–(C)). Means for RGR were positively related to RAG (*P* = 0·06) and log2(RAG:SAG) (*P* = 0·001) and negatively related to SAG (*P* = 0·02; Fig. 3(A)–(C)).

**Table 3.** Pilot study: glycaemic, insulinaemic and hunger responses

	Chew 1	Chew 2	Chew 3	Chew 4	Chew 5	Chew 6	P*
Glucose (mmol/l)							
iAUC 0–2 h^†^	142 ± 11^b^	159 ± 11^a^^b^	156 ± 13^a^^b^	162 ± 11^a^^b^	161 ± 11^a^^b^	180 ± 13^a^	0·014
iAUC 2–3 h^†^	12 ± 3	11 ± 2	11 ± 3	9 ± 2	12 ± 3	9 ± 2	0·61
iAUC 0–3 h^†^	154 ± 12	169 ± 12	167 ± 14	171 ± 13	174 ± 12	189 ± 13	0·089
2 h increment	0·17 ± 0·10	0·23 ± 0·09	0·20 ± 0·10	0·07 ± 0·10	0·15 ± 0·12	0·11 ± 0·11	0·65
Insulin (pmol/l)							
iAUC 0–2 h^‡^	111 ± 13^b^	120 ± 16^b^	117 ± 12^b^	144 ± 17^a^^b^	141 ± 16^a^^b^	167 ± 19^a^	<0·001
iAUC 2–3 h^‡^	12 ± 4	10 ± 4	11 ± 3	10 ± 3	13 ± 5	11 ± 4	0·95
iAUC 0–3 h^‡^	123 ± 14^b^	130 ± 19^b^	128 ± 13^b^	154 ± 19^a^^b^	154 ± 19^a^^b^	177 ± 21^a^	<0·001
Hunger (mm)							
tAUC 0–2 h^§^	84 ± 7^a^^b^	82 ± 7^a^^b^	86 ± 7^a^	82 ± 7^a^^b^	82 ± 7^a^^b^	74 ± 7^b^	0·042
tAUC 2–3 h^§^	58 ± 4	56 ± 4	58 ± 4	57 ± 4	61 ± 4	54 ± 4	0·083
tAUC 0–3 h^§^	142 ± 11^a^^b^	139 ± 10^a^^b^	144 ± 11^a^	139 ± 11^a^^b^	143 ± 10^a^^b^	127 ± 10^b^	0·030
2 h increment^¶^	−12·4 ± 4·7	−14·7 ± 4·2	−15·4 ± 4·2	−11·1 ± 4·1	−13·1 ± 3·9	−17·8 ± 4·2	0·23
3 h increment^¶^	3·8 ± 3·8	1·0 ± 4·0	3·0 ± 3·8	3·9 ± 3·8	4·2 ± 4·0	1·7 ± 4·0	0·84

Results are given as means±sem for *n* 40 subjects in each study except where otherwise indicated.iAUC, incremental areas under the curve; tAUC, total areas under the curve.

† Incremental area under the curve; units = mmol × min/l.

‡ Incremental area under the curve; units = pmol × h/l.

§ Total area under the curve; units = mm × h.

¶ Difference from fasting hunger rating; units = mm.

* Significance of heterogeneity among chews by analysis of variance (ANOVA).

^ab^Means with different letter superscripts differ significantly (*P* < 0·05 by Tukey's test).

Fasting insulin means(±sem) were similar among chews (*P* = 0·49) varying from 23 ± 2 (C4) to 27 ± 4 pmol/l (C2). Mean insulin increments differed among chews from 30 through 75 min (Fig. 2(D)). Insulin iAUC0-2 differed among chews (*F*_(5,195)_ = 6·48, *P* < 0·001) with C1, C2 and C3, respectively, less than C6 (Table 3). Means for RIR for C1 and C2, 68·7 ± 5·1 and 71·8 ± 4·6 %, respectively, were significantly less than that for C6, 100 ± 5·8 % (Fig. 2(D)). Means for iAUC2–3 h did not differ significantly, but insulin iAUC0-3 after C6 was significantly greater than those after C1–3 (Table 3). Means for insulin iAUC0–2 and iAUC0–3, respectively, were positively related to RAG (*P* = 0·03 and *P* = 0·03) and log2(RAG:SAG) (*P* = 0·008 and *P* = 0·006) and negatively to SAG (both *P* = 0·01; Supplementary Figs. S1(D)–(F) and S2(D)–(F)). Means for RIR were positively related to RAG (*P* = 0·008) and log2(RAG:SAG) (*P* = 0·003) and negatively related to SAG (*P* = 0·002; Fig. 3(D)–(F)).

**Fig. 4.**
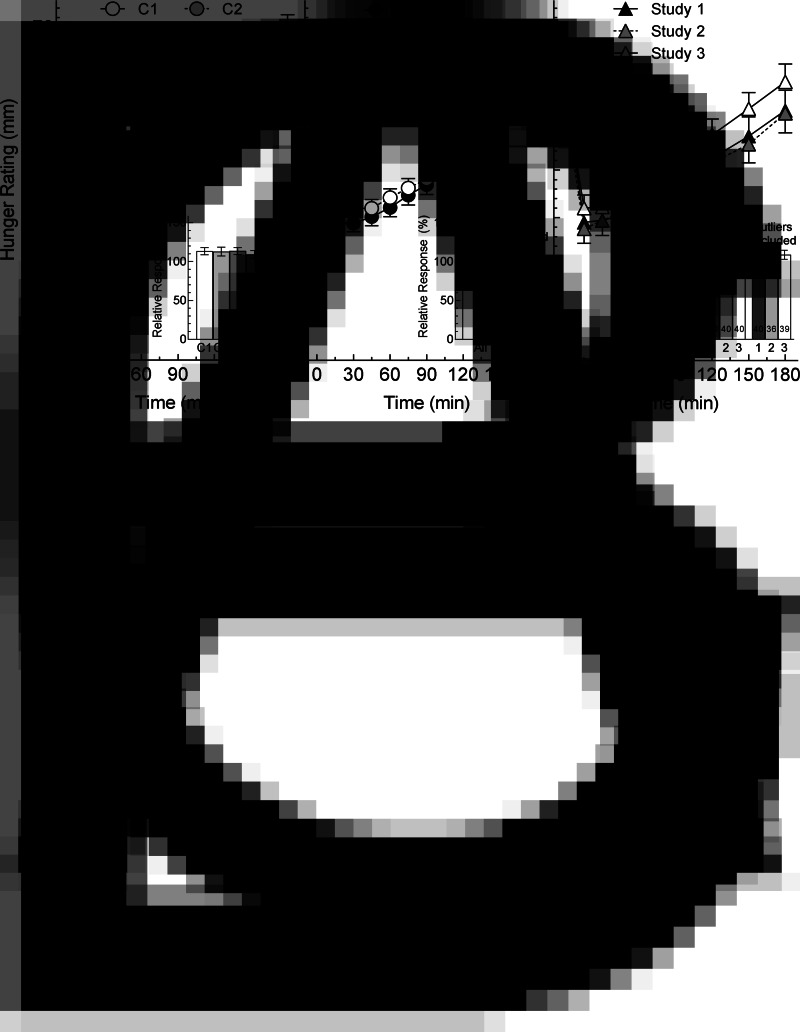
Subjective hunger responses. (A) Hunger ratings for six chews (pilot trial, means ± sem for *n* 40). Significance of differences by Tukey's test: a, C3vC6, *P* = 0·04; b, C3vC6, *P* = 0·08; c, C1vC6, *P* = 0·06; d, C3vC6, *P* = 0·06; all others *P* > 0·10. The inset shows tAUC0–2 for the six chews expressed as a percentage of C6 normalised so the mean for C6 = 100 (means ± sem, *n* 40). (B) Pooled hunger ratings for test and control granolas from the three granola studies (means ± sem for *n* 120). Significance of differences between test and control by Tukey's test: e, *P* = 0·03; all others *P* > 0·10. The inset shows tAUC0–2 for test granola expressed as a percentage of control before and after excluding outliers >2 × sd from the mean (means ± sem and *n*). (C) Mean hunger rating for test and control granola for the participants in each of the three studies (means ± sem for *n* 40). None of the differences are significant (*P* > 0·10). The inset shows tAUC0–2 for test granola expressed as a percentage of control before and after excluding outliers >2 × sd from the mean (means ± sem and *n*).

Hunger tAUC0–2 differed among chews (*F*_(5,195)_ 2·35, *P* = 0·04) with the only significant difference being that C3 was greater than C6 (Table 3). However, when expressed as RHR, the differences among chews were not significant (Fig. 4(A)). Means for hunger tAUC2-3 did not differ significantly, but the mean tAUC0–3 for C1 was significantly greater than that for C6 (Table 3). Hunger ratings among chews differed significantly at 45, 60, 90 and 105 min (Fig. 4(A)), but there was no significant difference at either 2 or 3 h (Table 3). Neither mean hunger tAUC0–2 nor tAUC0–3 were significantly related to RAG or SAG; log2(RAG:SAG) was significantly related to mean tAUC0–2 (*P* = 0·04) but not to tAUC0–3 (*P* = 0·10; Supplementary Figs. S1(G)–(I) and S2(G)–(I)). Means for RHR were negatively related to RAG (*P* = 0·15) and log2(RAG:SAG) (*P* = 0·02) and positively related to SAG (*P* = 0·07; Fig. 3(G)–(I)).

### Granola study end points

Means for glucose iAUC0–2 for TG1, TG2 and TG3 were significantly less than their respective CGs (*P* = 0·004, *P* = 0·002, *P* = 0·03, respectively, Table 4). When the values for the three studies were pooled, the difference between TG and CG was highly significant (*F*_(1,117)_  24·4, *P* < 0·001), with no difference between studies (*F*_(2,117)_= 0·02, *P* = 0·98) and no test meal × study interaction (*F*_(2,117)_  0·02, *P* = 0·98). The overall RGR for TG (mean, 99 % confidence interval) was 86·9 % (80·1, 93·7) (Fig. 2(B)) with no difference among studies (Fig. 2(C)). After removing four outliers, the RGR for TG (*n* 116) became 82·7 % (77·2, 88·1). There were no significant differences between TG and CG for glucose iAUC2–3, but the results for glucose iAUC0–3 were similar to those for iAUC0–2 (Table 4). Overall, glucose increment after TG was significantly less than CG at 15, 30, 45 and 60 min (Fig. 2(B)), but the differences at 2 h were not significant (Table 4). The mean glucose response curves for the three studies were virtually identical (Fig. 2(C)). Mean glucose iAUC0–2 for the six granolas were positively related to RAG (*P* = 0·01) and log2(RAG:SAG) (*P* < 0·001) and negatively to SAG (*P* < 0·001) with similar relationships for mean glucose iAUC0–3 (Supplementary Figs. S1(A)–(C) and S2(A)–(C)). Means for RGR were positively related to RAG (*P* = 0·02) and log2(RAG:SAG) (*P* = 0·001) and negatively related to SAG (*P* = 0·001; Fig. 3(A)–(C)).

**Table 4.** Granola studies: glycaemic, insulinaemic and hunger responses

	Study 1 (*n* 40)		Study 2 (*n* 40)		Study 3 (*n* 40)		Mean of three studies	
	CG1	TG1	CG2	TG2	CG3	TG3	CG	TG
Glucose (mmol/l)								
iAUC 0–2 h^a^	118 ± 8	98 ± 8^††^	118 ± 7	96 ± 8^††^	119 ± 9	99 ± 8^†^	118 ± 4	98 ± 4*
iAUC 2–3 h^a^	10 ± 2	8 ± 2	10 ± 2	8 ± 2	6 ± 2	6 ± 1	9 ± 1	7 ± 1
iAUC 0–3 h^a^	128 ± 8	106 ± 8^††^	128 ± 8	104 ± 9^††^	125 ± 9	105 ± 9^†^	127 ± 5	105 ± 5*
2 h increment	0·11 ± 0·07	0·09 ± 0·06	0·24 ± 0·07	0·11 ± 0·07	0·05 ± 0·07	0·13 ± 0·06	0·14 ± 0·04	0·11 ± 0·04
Insulin (pmol/l)								
iAUC 0–2 h^b^	147 ± 18	124 ± 16^†^	201 ± 23	161 ± 17^†^	205 ± 17	174 ± 16^†^	184 ± 11	153 ± 9*
iAUC 2–3 h^b^	12 ± 4	11 ± 5	12 ± 3	8 ± 2	13 ± 3	8 ± 1	12 ± 2	9 ± 2
iAUC 0–3 h^b^	159 ± 20	135 ± 19^†^	213 ± 24	169 ± 18^†^	218 ± 18	182 ± 16^†^	197 ± 12	162 ± 10*
Hunger (mm)								
tAUC 0–2 h^c^	70 ± 6	70 ± 6	65 ± 6	74 ± 6	75 ± 6	76 ± 5	70 ± 3	74 ± 3
tAUC 2–3 h^c^	47 ± 4	47 ± 4	45 ± 4	47 ± 4	53 ± 3	53 ± 3	48 ± 2	49 ± 2
tAUC 0–3 h^c^	117 ± 9	118 ± 9	110 ± 9	121 ± 10	128 ± 9	129 ± 8	118 ± 5	123 ± 5
2 h increment^d^	−14·2 ± 4·3	−11·7 ± 3·8	−11·2 ± 4·3	−10·0 ± 3·9	−9·1 ± 3·4	−6·6 ± 3·4	−11·5 ± 2·4	−9·4 ± 2·1
3 h increment^d^	−2·3 ± 3·9	−3·4 ± 3·6	−0·2 ± 4·8	−0·2 ± 4·4	2·5 ± 3·4	4·1 ± 3·7	−0·0 ± 2·4	0·2 ± 2·3

Results are given as means±sem for *n* 40 subjects in each study except where otherwise indicated.

CG, control granola; iAUC, incremental areas under the curve; tAUC, total areas under the curve; TG, test granola.

a Incremental area under the curve; units = mmol × min/l.

b Incremental area under the curve; units = pmol × h/l.

c Total area under the curve; units = mm × h.

d Difference from fasting hunger rating; units = mm.

Significant difference between test and control by *t*-test (†*P* < 0·05, ††*P* < 0·01).

Significant difference between test and control by analysis of variance (ANOVA) (*P ≤ 0·001).

Means insulin iAUC0–2 for TG1, TG2 and TG3 were significantly less than their respective CGs (*P* = 0·01, *P* = 0·02 and *P* = 0·045, respectively, Table 4). When the values for the three studies were pooled, the difference between TG and CG was highly significant (*F*_(1,117)_=  20·8, *P* < 0·001). Participants in studies 2 and 3 had higher insulin responses than those in study 1 (*F*_(2,117)_ =  3·18, *P* = 0·045) (Table 3 and Fig. 2(F)), but there was no significant test meal × study interaction (*F*_(2,117)_ =  0·46, *P* = 0·63). Overall, the RIR for TG did not differ significantly from 100, being (mean, 95 % confidence interval) 95·7 % (85·8, 105·6), Fig. 2(E)) with no difference in RIR among studies (Fig. 2(F)). After removing six outliers, the RIR for TG (*n* 114), 87·3 % (79·7, 94·9), was significantly less than 100 and not significantly different from mean RGR. There were no significant differences for insulin iAUC2–3 (Table 4). The results for insulin iAUC0–3 were similar to those for iAUC0–2 (Table 4), but there was no significant difference among studies (*P* = 0·08) and no test meal × study interaction (*P* = 0·55). Mean insulin iAUC0–2 for the six granolas were not significantly related to RAG, SAG or log2(RAG:SAG) (Supplementary Fig. S1(D)–(F)) and neither was insulin iAUC0–3 (Supplementary Fig. S2(D)–(F)). Means for RIR were positively related to RAG (*P* = 0·03) and log2(RAG:SAG) (*P* = 0·003) and negatively related to SAG (*P* = 0·003; Fig. 3(D)–(F)).

There were no significant differences in mean hunger tAUC0–2 between TG and CG in any of the three studies (Table 4). When the results were pooled, there was no significant difference between TG and CG (*F*_(1,117)_ = 1·79, *P* = 0·18), no difference among the three studies (*F*_(2,117)_ = 0·36, *P* = 0·70) and no test meal × study interaction (*F*_(2,117)_ = 1·05, *P* = 0·35) (Table 4). There were also no significant differences for hunger tAUC2–3 or tAUC0–3 (Table 4). When the results were pooled, the only significant difference in mean hunger rating was at 30 min, with TG > CG (Fig. 4(B)). There were no significant differences in hunger increments at either 2 or 3 h (Table 4) and no significant correlation between mean hunger tAUC0–2 and tAUC0–3 (Supplementary Figs. S1(G)–(I) and S2(G)–(I)] for the six granolas. Means for RHR were negatively related to RAG (*P* = 0·02) and log2(RAG:SAG) (*P* = 0·02) and positively related to SAG (*P* = 0·18; Fig. 3(G)–(I)).

### Comparison of granola and pilot study results

The slopes of seventeen of the eighteen regression lines for the glucose, insulin and hunger iAUCs on RAG, SAG and log2(RAG:SAG) for the pilot study data did not differ significantly from those for the granola study data; however, the elevations or intercepts of these regression lines differed significantly for the glucose and hunger iAUCs on RAG, SAG and log2(RAG:SAG) and for insulin iAUC on RAG (Supplementary Figs. S1(A)–(I) and S2(A)–(I) and Table S3). However, the slopes and *y*-intercepts (mean ± sem) of the regression lines for RGR on log2(RAG:SAG) for the pilot data, 4·03 ± 1·11 and 77·7 ± 3·5, respectively, were similar to those for the granola data, 4·71 ± 0·58 (*P* = 0·59) and 72·1 ± 2·6 (*P* = 0·12; Fig. 3(C) and Supplementary Table S3). The only significant differences between the pilot and granola data were the regression line slopes for RGR on SAG, RIR on RAG and RIR on log2(RAG:SAG) (Fig. 3(B), (D) and (F)) and the elevations for RHR on RAG (Fig. 3(G) and Supplementary Table S4).

### Granola studies: exploratory multiple regression analysis

Stepwise multiple regression analysis (SWMRA) showed that glucose iAUC0–2 (model *r*^2^ 0·419) and iAUC0–3 (model *r*^2^ 0·403) were positively associated with CG (*v*. TG), non-Caucasian ethnicity and age and negatively with height and fasting glucose. Glucose iAUC2–3 (model *r*^2^ 0·099) and inc120 (model *r*^2^ 0·126) were positively associated with non-Caucasian ethnicity and age and negatively with fasting glucose. However, the only significant determinant of RGR was test meal (CG > TG; model *r*^2^ 0·057; Supplementary Table S5).

The SWMRA showed that insulin iAUC0–2 (model *r*^2^ 0·419) and iAUC0–3 (model *r*^2^ 0·403) were positively associated with the analogous glucose response (glucose iAUC0–2 or iAUC0–3, respectively), CG (*v*. TG), female sex, non-Caucasian ethnicity, BMI and fasting insulin and negatively with age. Insulin iAUC2–3 model (*r*^2^ 0·321) was positively associated with glucose iAUC2–3, BMI and fasting glucose and negatively with age and weight. Insulin inc120 min (model *r*^2^ 0·329) was positively associated with glucose inc120 min, CG (*v*. TG), female sex, non-Caucasian ethnicity, BMI and fasting insulin and negatively with age and height. However, the only significant determinants of RIR were RGR and fasting glucose (model *r*^2^ 0·193, Supplementary Table S6).

The SWMRA showed that hunger tAUC0–2 (model *r*^2^ 0·075), tAUC2–3 (model *r*^2^ 0·034), tAUC0–3 (model *r*^2^ 0·069) and inc120 min (model *r*^2^ 0·050) were higher in females; in addition, hunger tAUC0–2 was negatively related to glucose iAUC0–2, hunger tAUC0–3 was positively related to fasting insulin and hunger inc120 min was higher in non-Caucasians. Hunger inc180 min was higher in non-Caucasians (model *r*^2^ 0·021; Supplementary Table S7).

## Discussion

The results showed that reducing the RAG:SAG ratio in granola cereal significantly reduced postprandial glucose and insulin responses but had no effect on subjective hunger and did not significantly delay the return of glucose or hunger to baseline. The mean ± sem RGR for TG, 82·7 ± 2·7, was similar to the value of 85·3 ± 4·0 predicted from the results of the pilot study. This prediction was derived as follows: the slope of the regression of RGR on log2(RAG:SAG) in the pilot study, 4·03 ± 1·11, means that each one unit decrease in log2(RAG:SAG) (i.e., each 50 % reduction of RAG:SAG) leads to a 4·03 ± 1·11 reduction in RGR. The difference between log2 of the mean RAG:SAG ratios for CG (log2(60·9) = 5·93) and TG (log2(4·9) = 2·29) is 3·64, a difference that is be expected to reduce RGR by 3·64 × 4·03 ± 1·11 or 14·7 ± 4·0.

Our findings are consistent with those in the literature, showing that the RAG content of foods is positively, and SAG content is negatively, associated with glucose and insulin responses^(9,26–31,39)^. We used the RAG:SAG ratio as our main outcome because RAG and SAG alone measure both the quality and the quantity of carbohydrate, each of which influences glycaemic responses, whereas the RAG:SAG ratio is independent of the amount of carbohydrate. We used log2(RAG:SAG) because the slope of the regression line represents the change in the end point for every doubling (or halving) of RAG:SAG. To compare the present results with those in the literature, we compared the regressions of RGR on RAG, SAG and log2(RAG:SAG) for five different data sets: Englyst99^(26)^, Englyst03^(27)^, Garsetti^(9)^, three French studies^(28,29,39)^ and two PepsiCo studies (the present study and^(30)^). The slopes of the regressions of RGR on RAG and RGR on SAG did not differ significantly among the data sets (Supplementary Figs. S3(A) and (B), and Table S8), but the slope of RGR on log2(RAG:SAG) was significantly greater for Englyst03^(27)^ *v*. the PepsiCo studies (Supplementary Fig. S3(C) and Table S8). However, since adding fat to carbohydrate reduces the glycaemic response^(40)^, the effect of RAG:SAG in Englyst03^(27)^ was confounded by the variable fat content (0·4–13·2 g) of the 50 g avCHO portions of the twenty-three foods tested. Multiple regression analysis of the Englyst03 data showed that RGR was independently associated with both fat (*P* = 0·03) and log2(RAG:SAG) (*P* = 0·001). Including fat in the model reduced the slope of RGR on log2(RAG:SAG) from 9·98 to 7·14, a value no longer significantly different from the other four data sets (Supplementary Tables S8 and S9). Furthermore, the slope of RGR on fat, −1·51 indicates that the 12·8 g range of fat in Englyst03 reduced RGR by 19·3 %, a value similar to the 18·0 [10·4, 24·7]% (mean [95 % confidence interval]) reduction predicted for adding 12·8 g fat to carbohydrate from our recent meta-analysis^(40)^. Protein did not confound the effect of RAG:SAG in Englyst03. In Garsetti^(9)^ (the only other study with enough foods to allow multiple regression analysis), protein, but not fat, had a significant effect on RGR when combined with log2(RAG:SAG). However, including protein in the model did not meaningfully change the slope of RGR on log2(RAG:SAG) (Supplementary Table S8).

An important finding was that the TGs reduced mean insulin iAUC0–2 relative to CG by (mean [95 % CI]) 12·7 % [5·1, 20·3], a value not significantly different from the percentage reduction in glucose iAUC0–2, 17·3 % [11·9, 22·8] (*P* = 0·32). This suggests that the lower glycaemic response elicited by TG *v*. CG was not due to an increased insulin response.

We analyzed the combined data from the three granola studies to see whether the results obtained differed according to the age, sex, ethnicity or other characteristics of the subjects. Glucose iAUC was lower in Caucasian *v*. other ethnicities, was positively related to age and was negatively related to height and fasting glucose; however, neither ethnicity, sex, age, height, weight, BMI, fasting glucose nor fasting insulin was a significant determinant of RGR (Supplementary Table S5). A possible explanation for this is that such subject factors affect absolute glycaemic responses by influencing the metabolism of glucose after it has been absorbed from the small intestine. However, such effects would influence the glycaemic response elicited by any test meal to the same extent and, thus, affect absolute responses but not RGR. This is consistent with studies showing that mean glucose iAUC values were significantly lower in subjects ≤40 *v*. >40 year of age^(41)^ and significantly lower in Caucasian *v*. other ethnicities^(41,42)^, whereas there was no significant effect of age or ethnicity^(41,42)^ on glycaemic index (GI), a specific example of an RGR. Any subject factor able to affect RGR would have to influence the rate of glucose absorption from either the test or the control food, but not both. For example, the GI values of nine non-dairy foods tested in rural Africans^(43)^ and Europeans^(1)^ correlated almost perfectly (*r* 0·969, *P* < 0·001, mean difference 0·9, Fig. 2: 10C in^(7)^), but milk had a lower GI in Africans and Europeans (3 *v*. 34) presumably due to the high prevalence of low intestinal lactase activity in Africans. Rice may have a higher GI in non-Caucasians than in Caucasians^(44)^, an effect that may be attributed to Chinese chewing rice into smaller particle sizes than Whites do^(45)^. In both these cases, genetic or cultural factors linked to ethnicity influenced the rate of absorption of the carbohydrates in the test food, but not the reference food, glucose.

The objectives of the present study were based on the overall hypothesis that reducing the RAG:SAG ratio provides more slowly released carbohydrate, thereby eliciting lower glucose and insulin responses, providing steadier energy to the body and delayed return of hunger sensations after eating. The results showed that, despite the large difference in the RAG:SAG ratio between TG and CG, there was no significant difference in glucose or hunger increments at 2 h. However, glucose increment at 2 h may not be a reliable marker of slow glucose absorption because any such relationship may be confounded by subject characteristics. The present results show that the glucose increment at 2 h was negatively associated with non-Caucasian ethnicity and fasting glucose and positively with age (Supplementary Table S5).

Postprandial hunger ratings did not differ between TG and CG, but this does not necessarily rule out an effect on food intake, since Anderson *et al.* found that, 2 h after consumption of test meals high in resistant starch, food intake was significantly lower than after consumption of test meals high in rapidly digested starch^(31)^, despite no significant difference in subjective appetite.

In summary, the results showed that reducing the RAG:SAG ratio in chews or granolas significantly reduced postprandial glucose and insulin responses. Compared with conventional granolas with RAG:SAG ratios >54, test granolas with RAG:SAG ratios <5·5 reduced glucose iAUC by 17–18 %, a difference similar to the 15 % reduction predicted by the pilot trial using chews. The reduction in the relative glucose response elicited by each 50 % reduction in the RAG:SAG ratio in the present studies did not differ significantly from those in the literature. However, compared with control granola, test granola did not reduce postprandial hunger or delay the return of glucose or hunger to baseline.

## Supplementary material

For supplementary material accompanying this paper visit https://doi.org/10.1017/jns.2022.22. click here to view supplementary material
