# Supplementary material for: Decreasing the RAG:SAG ratio of granola cereal predictably reduces postprandial glucose and insulin responses: a report of four randomised trials in healthy adults
Source: J Nutr Sci. 2022 Mar 17;11:e21. doi: 10.1017/jns.2022.22 (PMC8943571; doi:10.1017/jns.2022.22)
Supplement: Supplementary file 1 [file S2048679022000222sup001.docx]

Table of Contents

[Supplementary Methods 2](#_Toc79574710)

[Inclusion/Exclusion Criteria 2](#_Toc79574711)

[Glucose and insulin analysis 3](#_Toc79574712)

[Supplementary Table 1. RAG and SAG Analysis of Test Chews 5](#_Toc79574713)

[Supplementary Table 2: Supplement and medication use. 6](#_Toc79574714)

[Supplementary Results 8](#_Toc79574715)

[Protocol deviations 8](#_Toc79574716)

[Adverse events 8](#_Toc79574717)

[Supplementary Figure 1: Associations between AIC0-2h and RAG, SAG and log2(RAG:SAG). 9](#_Toc79574718)

[Supplementary Figure 2: Associations between AIC0-3h and RAG, SAG and log2(RAG:SAG). 10](#_Toc79574719)

[Supplementary Table 3: Regressions of relative glucose/insulin responses on RAG, SAG and log2(RAG:SAG): Pilot vs Granola data. 11](#_Toc79574720)

[Supplementary Table 4: Multiple regression analysis for Glucose Responses 12](#_Toc79574721)

[Supplementary Table 5: Multiple regression analysis for Insulin Responses 13](#_Toc79574722)

[Supplementary Table 6: Multiple regression analysis for Hunger responses. 14](#_Toc79574723)

[Supplementary Figure 3: Associations between relative glucose/insulin responses and RAG, SAG and log2(RAG:SAG): comparison with literature data. 15](#_Toc79574724)

[Supplementary Table 7: Regressions of relative glucose/insulin responses on RAG, SAG and log2(RAG:SAG): comparison with literature data. 16](#_Toc79574725)

[Supplementary Table 8: Multiple regressions of fat, protein and RGR/RIR on log2(RAG:SAG) for Englyst03 and Garsetti datasets. 17](#_Toc79574726)

[Supplementary References 18](#_Toc79574727)

# Supplementary Methods

## Inclusion/Exclusion Criteria

The following participant inclusion/exclusion criteria were the same for all 4 studies.

**Inclusion Criteria**

- Male or non-pregnant, non-lactating females, 18-65 years of age, inclusive

- Body mass index (BMI) between 21.00 and 32.00 kg/m², inclusive, at screening (visit 1).

- No participation in a PepsiCo study at INQUIS for at least 1 year from signing the consent form.*

- No participation in any clinical trial for at least 30 days from signing the consent form.*

- Willing to maintain habitual diet, physical activity pattern, and body weight throughout the trial and to refrain from smoking for 12hr prior to each visit.

- Willing to maintain current dietary supplement use throughout the trial. On test days, subject agrees not to take any dietary supplements until dismissal from INQUIS. Failure to comply will result in a rescheduled test visit.

- Fasting serum glucose <7.0 mmol/L or capillary whole blood glucose <6.3 mmol/L.

- Willing to abstain from alcohol and avoid vigorous physical activity for 24 h prior to all test visits.

- Absence of health conditions that would prevent fulfillment of study requirements as judged by the Investigator on the basis of medical history.

- Understanding the study procedures and willing to provide informed consent to participate in the study and authorization to release relevant protected health information to the study investigator.

**Exclusion Criteria**

- Failure to meet any one of the inclusion criteria

- Known history of AIDS, hepatitis, or presence of clinically important endocrine (including Type 1 or Type 2 diabetes mellitus), cardiovascular (including, but not limited to, atherosclerotic disease, history of myocardial infarction, peripheral arterial disease, stroke), pulmonary, biliary or GI disorders.

- Use of medications known to influence carbohydrate metabolism, including, but not limited to adrenergic blockers, diuretics, thiazolidinediones, metformin and systemic corticosteroids within 4 weeks of screening, or with any condition which might, in the opinion of the medical directorof INQUIS either: 1) make participation dangerous to the subject or to others, or 2) affect the results.

- Major trauma or surgical event within 3 months of screening.

- Unwillingness or inability to comply with the experimental procedures and to follow INQUIS safety guidelines.

- Lactose intolerance.

- Known intolerance, sensitivity or allergy to any ingredients in the study products.

- Extreme dietary habits, as judged by the Investigator (i.e. Atkins diet, very high protein diets, etc).

- Uncontrolled hypertension (systolic blood pressure ≥160 mm Hg or diastolic blood pressure ≥100 mm Hg as defined by the average blood pressure measured at screening.

- Change in body weight of >3.5kg within 4 weeks of the screening visit.

- Presence of any signs or symptoms of active infection within 5 d prior to any test visit. If an infection occurs during the study period, test visits should be rescheduled until all signs and symptoms have resolved and any treatment (i.e. antibiotic therapy) completed at least 5 d prior to each test visit.

- History of cancer in the prior two years, except for non-melanoma skin cancer.

- Recent history (within 12 mo of screening) or strong potential for alcohol or substance abuse. Alcohol abuse is defined as >14 drinks per week (1 drink=12 oz beer, 5 oz wine, or 1.5 oz distilled spirits).

- Exposure to any investigational drug product within 30 g prior to screening.

* Criteria added for the 3 granola studies.

## Glucose and insulin analysis

Glucose was measured twice in each 0 min sample and analytical coefficient of variation (CV = 100×SD/mean) was calculated from the SD determined as follows: D = √ [Σd^2^)/2n], where d = the difference between duplicate measures and n = the number of blood samples measured in duplicate.

Pilot Trial:

Values for glucose were missing for 8 of the 1920 samples (0.4%) due to clotted blood. Missing values were imputed as follows: 1 missing -5 min value was replaced by the value at 0 min, 3 missing values at 60 min and 1 at each of 90 and 120 min were replaced by the mean of surrounding values and 1 missing value at each of 30 and 45 min was replaced using a procedure described by Snedecor and Cochran (^[[1]](#endnote-2)^) since these times were near the peak and values derived from the Snedecor and Cochran procedure were greater than the mean of the surrounding values.

Mean±SD glucose in the 126 fasting samples for which duplicates could be measured was 4.51±0.0650 mmol/L yielding an analytical CV of 1.4%. Mean±SD glucose in the 139 -5 and 0 min samples was 4.53±0.127 mmol/L for a CV of 2.8%, which is greater than analytic variation because it reflects both analytical variation and minute-to-minute variation.

There were no missing samples for insulin analysis, but 1245 (43%) of the 2880 samples analyzed had undetectable concentrations (<3 μU/mL) and were ascribed a value of 2.99 μU/mL for statistical analysis; 818 (66%) of these values occurred at -5, 0, 120, 150 or 180min, 312 (25%) between 75 and 105 min and 708 (57%) occurred in 15 subjects. The number of undetectable values did not differ significantly among chews (range 201-216, p=0.96). Mean±SD insulin in the 80 -5 and 0min samples was 25.4±11.5 pmol/L for a CV of 45.3% which includes both analytical and minute-to-minute variation in insulin which is secreted in a pulsatile fashion with an amplitude of ±0.5μU/mL about the mean and frequency of ~13 min (^[[2]](#endnote-3)^).

Granola Studies:

Study 1: Values for glucose were missing for 8 of the 960 samples (0.8%) due to clotted blood. Mean±SD glucose in the 71 fasting samples for which duplicates could be measured was 4.38±0.0713 mmol/L for a CV of 1.6%. Mean±SD glucose in the 78 -5 and 0 min samples was 4.39±0.113 mmol/L for a CV of 2.6%.

No samples were missing for insulin, but 406 (42%) of the 960 samples had undetectable insulin (<3 μU/mL) and were ascribed a value of 2.99 μU/mL for statistical analysis; 283 (70%) of these values occurred at -5, 0, 120, 150 or 180min, 84 (21%) between 75 and 105 min and 239 (59%) occurred in 15 subjects. The number of undetectable values did not differ significantly between CG1 (205, 45%) and TG1 (201, 41%). Mean±SD insulin in the 80 -5 and 0min samples was 22.4±8.46 pmol/L for a CV of 37.9% which includes both analytical and minute-to-minute variation of insulin which is secreted in a pulsatile fashion with an amplitude of ±0.5μU/mL about the mean and frequency of ~13 min (2).

Study 2: Values for glucose were missing for 8 of the 960 samples (0.8%) due to clotted blood. Mean±SD glucose in the 70 fasting samples for which duplicates could be measured was 4.44±0.0480 mmol/L for a CV of 1.1%. Mean±SD glucose in the 76 -5 and 0 min samples was 4.45±0.113 mmol/L for a CV of 2.5%.

No samples were missing for insulin, but 339 (35%) of the 960 samples had undetectable insulin (<3 μU/mL) and were ascribed a value of 2.99 μU/mL for statistical analysis; 250 (74%) of these values occurred at -5, 0, 120, 150 or 180min, 64 (19%) between 75 and 105 min and 211 (62%) occurred in 15 subjects. The number of undetectable values did not differ significantly between CG2 (168, 50%) and TG2 (171, 50%). Mean±SD insulin in the 80 -5 and 0min samples was 27.7±15.1 pmol/L for a CV of 54.6% which includes both analytical and minute-to-minute variation of insulin which is secreted in a pulsatile fashion with an amplitude of ±0.5μU/mL about the mean and frequency of ~13 min (2).

Study 3: Values for glucose were missing for 4 of the 960 samples (0.4%) due to clotted blood. The mean±SD glucose concentration in the 68 fasting (0 min) samples for which duplicates could be measured was 4.34±0.0429 mmol/L for a CV of 1.0%. The mean±SD glucose concentration in the 77 -5 and 0 min samples was 4.34±0.168 mmol/L for a CV of 3.9%.

No samples were missing for insulin, but 71 (7.4%) of the 960 samples had undetectable insulin (<3 μU/mL) and were ascribed a value of 2.99 μU/mL for statistical analysis; 62 (87%) of these values occurred at -5, 0, 120, 150 or 180min, all but one of the rest, n=8 (11%), between 75 and 105 min and 57 (80%) occurred in 13 subjects. Significantly more undetectable values occurred for TG3 (n=23, 32%) compared to CG (n=48, 68%), p=0.003. Mean±SD insulin in the 80 -5 and 0min samples was 39.6±11.6 pmol/L for a CV of 29.4% which includes both analytical and minute-to-minute variation of insulin which is secreted in a pulsatile fashion with an amplitude of ±0.5μU/mL about the mean and frequency of ~13 min (2).

Missing values for glucose at either -5 or 0 min were replaced by the other existing fasting value, missing values at 15-150 min were imputed as the mean of surrounding values, and missing values at 180 min were imputed using a procedure described by Snedecor and Cochran (1).

### Supplementary Table 1. RAG and SAG Analysis of Test Chews

| Test Meal | Abbr. | Starch | Batch | | | Mean for Chews | RAG:SAG Ratio |
| --- | --- | --- | --- | --- | --- | --- | --- |
|  |  |  | 1 | 2 | 3 |  |  |
| Chew 427.0 (control) | C40-0.1 | RAG | 41.77±0.41 | 39.34±0.26 | 40.55±1.56 | 40.55±1.22^b^ | 42.8 |
|  |  | SAG | 0.16±0.08 | 0.71±0.86 | 1.98±0.40 | 0.95±0.94^d^ |  |
| Chew 10.8 | C40-4 | RAG | 41.18±0.44 | 39.28±0.81 | 40.23±0.38 | 40.23±0.95^bc^ | 13.4 |
|  |  | SAG | 2.42±0.93 | 4.02±1.42 | 2.60±0.25 | 3.01±0.87^c^ |  |
| Chew 5.5 | C40-8 | RAG | 40.99±0.52 | 40.78±0.46 | 41.99±0.26 | 41.25±0.65^b^ | 4.62 |
|  |  | SAG | 9.85±0.46 | 7.86±0.71 | 9.09±0.23 | 8.93±1.00^b^ |  |
| Chew 2.6 | C40-15 | RAG | 38.81±0.14 | 39.86±0.66 | 37.66±0.66 | 38.78±1.10^c^ | 2.38 |
|  |  | SAG | 15.86±0.29 | 15.98±0.66 | 17.07±1.28 | 16.31±0.67^a^ |  |
| Chew 4.1 | C30-8 | RAG | 31.52±0.21 | 29.80±0.16 | 30.41±0.18 | 30.58±0.87^d^ | 3.15 |
|  |  | SAG | 9.72±0.53 | 8.92±1.20 | 10.51±0.47 | 9.72±0.79^b^ |  |
| Chew 6.8 | C50-8 | RAG | 50.83±0.20 | 49.84±0.02 | 49.47±0.16 | 50.04±0.71^a^ | 5.49 |
|  |  | SAG | 8.68±0.13 | 8.06±0.21 | 10.62±0.73 | 9.12±1.34^b^ |  |
| Mean for Batches |  | RAG | 40.85±6.19 | 39.81±6.36 | 40.05±6.19 |  |  |
|  |  | SAG | 7.78±5.67 | 7.59±5.15 | 8.65±5.65 |  |  |

Values are means±SD for 2-4 determinations expressed as % (g/100g).

Abbr = abbreviation; RAG = Rapidly available glucose; SAG = slowly available glucose. RAG and SAG values are the means of the analysis of the 3 batches produced.

### Supplementary Table 2: Supplement and medication use.

| **Study*** | **Supplement / Medication** | **Dose** | **Units** | **Route** | **Frequency** |
| --- | --- | --- | --- | --- | --- |
| Pilot | Citalopram | 20 | mg | Oral | Once Daily |
| Pilot | Venlafaxine | 300 | mg | Oral | Once Daily |
| Pilot | Omega-3 | 1000 | mg | Oral | Once Daily |
|  | Vitamin D | 1000 | IU | Oral | Once Daily |
| Pilot | Calcium/Magnesium | 250 | mg | Oral | Once Daily |
|  | Omega-3 | 1 | teaspoon | Oral | Once Daily |
| Pilot | Multivitamin | 3 | capsules | Oral | Once Daily |
| Pilot | Vitamin B12 | 500 | mcg | Oral | Once Daily |
|  | Collagen 30 | 2.5 | mg | Oral | Once Daily |
| Pilot | Clonazepam | 0.5 | mg | Oral | Once Daily |
| Pilot | Tricira (birth control) | 1 | capsule | Oral | Once Daily |
| Pilot | Trandolapril | 2 | mg | Oral | Once Daily |
| Pilot | Vitamin D | 1000 | IU | Oral | Once Daily |
| Granola 1 | Ethinyl estradiol / levonorgestrel | 1 | capsule | Oral | Once daily |
| Granola 1 | Bupropion | 150 | mg | Oral | Once daily |
| Granola 1 | Venlafaxine | 150 | mg | Oral | Once daily |
|  | Vitamin B12, Vitamin D, Iron | 1000, 1000, 1 | mcg, IU, capsule | Oral | Once daily |
|  | Omega 3 fatty acids | 1 | capsule | Oral | Twice daily |
| Granola 1 | Omega 3 fatty acids | 1000 | mg | Oral | Once daily |
|  | Vitamin D, Vitamin B12 | 1000, 500 | IU, mcg | Oral | Once daily |
| Granola 1 | Fluticasone | 50 | mcg | Inhaled | As needed |
| Granola 1 | Vitamin B complex | 250 | Mg | Oral | Once daily |
|  | Collagen | 500 | Mg | Oral | Once daily |
| Granola 2 | Omega 3 fatty acids | 2400 | mg | Oral | Once daily |
|  | Vitamin D, Vitamin B12, magnesium | 50, 1200, 200 | mcg, mcg, mg | Oral | Once daily |
| Granola 2 | Multivitamin | 1 | cap | Oral | Once daily |
| Granola 2 | Tamsulosin | 0.4 | mg | Oral | Once daily |
|  | APO-Meloxicam | 7.5 | mg | Oral | Once daily |
| Granola 2 | Cipralex | 20 | mg | Oral | Once daily |
| Granola 2 | Multivitamin | 1 | cap | Oral | Once daily |
|  | Synthroid | 0.75 | mg | Oral | Once daily |
| Granola 2 | Norvasc | 2.5 | mg | Oral | Once daily |
| Granola 2 | Tricyclen Low | 1 | cap | Oral | Once daily |
| Granola 2 | Multivitamin | 2 | tab | Oral | Once daily |
| Granola 2 | Trazadone | 100 | mg | Oral | Once daily |
|  | Multivitamin | 1 | cap | Oral | Once weekly |
|  | Zopiclone | 5 | mg | Oral | Once daily |
| Granola 2 | Tricyclen Low | 1 | cap | Oral | Once daily |
| Granola 2 | Xarelto | 20 | mg | Oral | Once daily |
| Granola 2 | Nimodipine | 10 | mg | Oral | Once daily |
| Granola 2 | Sertraline | 50 | mg | Oral | Once daily |
| Granola 2 | Birth Control | 1 | tab | Oral | Once daily |

* Each box in this column represents 1 participant (some participants on > 1 medication/supplement).

**Supplementary Table 2 (Cont.): Supplement and medication use.**

| **Study*** | **Supplement / Medication** | **Dose** | **Units** | **Route** | **Frequency** |
| --- | --- | --- | --- | --- | --- |
| Granola 3 | Vitamin D | 2000 | IU | Oral | Once daily |
| Granola 3 | Vitamin C, multivitamin | 500, 1 | Mg, tablet | Oral | Once daily |
| Granola 3 | Tumeric/Curcumin/Ginger | 450/7.5/200 | mg | Oral | Once daily |
| Granola 3 | Vitamin D | 1000 | IU | Oral | Once daily |
| Granola 3 | Jameson Multivitamin | 1 | tab | Oral | Once daily |
| Granola 3 | Escitalopram | 10 | mg | Oral | Once daily |
| Granola 3 | Adderall XR | 20 | mg | Oral | Once daily |
|  | Contraceptive (Loestrin) | 1 | tab | Oral | Once daily |
| Granola 3 | Sertraline | 75 | mg | Oral | Once daily |
| Granola 3 | Contraceptive (Demulen 28) | 1 | cap | Oral | Once daily |
| Granola 3 | Contraceptive (Tricerra) | 1 | cap | Oral | Once daily |
| Granola 3 | Naproxin/esomeprazole | 500 | mg | Oral | Twice daily |
|  | Ibuprofen | 400 | mg | Oral | As needed |
|  | Vitamin D | 1000 | iu | Oral | Once daily |
|  | Tumeric | 450 | mg | Oral | Once daily |

* Each box in this column represents 1 participant (some participants on > 1 medication/supplement).

# Supplementary Results

## Protocol deviations

Pilot Trial: There were 35 protocol deviations; 31 of these consisted of subjects taking longer than 10 min to complete the test meal (29 subjects took between 10-15min, 1 took 16 min and 1 took 20 min). There was 1 missed VAS question, 1 late blood sample, 1 test meal repeated due to non-fasting and 1 interval of 18 days between test meals. None of these were considered to be serious deviations.

Granola Study 1: 5 participants took 10.5 to 12 min (longer than 10 min) to complete the test-meal on a total of 6 occasions. None of these were considered to be serious deviations.

Granola Study 2: 3 participants took 11 to 12 min to complete the test-meal on a total of 3 occasions. None of these were considered to be serious deviations.

Granola Study 3: 1 participant came in for first visit with a fasting glucose > 7.0 mmol/L; they initially claimed to be fasting, but when told they should see their doctor about it, they admitted they did not fast. The participant was withdrawn from the study.

## Adverse events

Pilot Trial: No adverse events were reported.

Granola Study 1: There was 1 adverse event (not serious) during the trial; upon attending for the second test, ID #6 had a mild headache which was not related to the study treatment and resolved without treatment; because of this, the second test was postponed for a week.

Granola Study 2: There was 1 adverse event (not serious) during the trial; upon attending for the first test one subject vomited after the fasting finger-prick blood samples and before consuming the test meal. The test was discontinued and the subject dropped out.

Granola Study 3: One subject fainted while having blood pressure measured during the screening visit. This isolated event was not related to the study product and the outcome was that the subject was not enrolled in the study.

### Supplementary Figure 1: Associations between AIC0-2h and RAG, SAG and log2(RAG:SAG).

Values are means±SEM for n=40 subjects. Circles show results for the Pilot study; black, grey and white triangles, respectively, show results for granola studies 1, 2 and 3. Lines are regression lines for the Pilot study (solid) and granola studies (dashed). Correlation coefficients (r) and P-values are give for the Pilot and Granola study data. The slope of the regression line for the pilot data does not differ significantly from that for the granola data in any of the panels. However, the elevation or intercept of the regression line for pilot data differs significantly from that for the granola data in panels A, B, C, D, G, H and I.

### Supplementary Figure 2: Associations between AIC0-3h and RAG, SAG and log2(RAG:SAG).

Values are means±SEM for n=40 subjects. Circles show results for the Pilot study; black, grey and white triangles, respectively, show results for granola studies 1, 2 and 3. Lines are regression lines for the Pilot study (solid) and granola studies (dashed). Correlation coefficients (r) and P-values are give for the Pilot and Granola study data. The slope of the regression line for the pilot data differs significantly from that for the granola data in panel B, but not in any of the other panels. The elevation or intercept of the regression line for pilot data differs significantly from that for the granola data in panels A, C, D, G, H and I.

### Supplementary Table 3: Regressions of AUCs for glucose, insulin and hunger on RAG, SAG and log2(RAG:SAG): Pilot vs Granola data.

| Regression | Slope | | | Elevation or y-intercept* | | |
| --- | --- | --- | --- | --- | --- | --- |
|  | Pilot | Granola | P | Pilot | Granola | P |
| GiAUC 0-2h on RAG | 2.99±1.16 | 1.61±0.36 | 0.23 | 76.3±32.6 | 66.2±9.6 | <0.001 |
| GiAUC 0-2h on SAG | -3.21±0.81 | -5.19±0.51 | 0.13 | 177±5.04 | 120±1.56 | <0.001 |
| GiAUC 0-2h on log2(R:S) | 7.08±1.71 | 5.52±0.34 | 0.36 | 140±5.37 | 85.3±1.53 | <0.001 |
| GiAUC 0-3h on RAG | 2.87±0.98 | 1.80±0.29 | 0.27 | 90.3±27.5 | 69.2±7.80 | <0.001 |
| GiAUC 0-3h on SAG | -3.03±0.64 | -5.49±0.70 | 0.047 | 187±4.01 | 129±2.16 | - |
| GiAUC 0-3h on log2(R:S) | 6.65±1.40 | 5.93±0.37 | 0.60 | 152±4.39 | 91.6±1.64 | <0.001 |
| IiAUC 0-2h on RAG | 5.49±1.73 | 1.63±2.34 | 0.35 | -20.3±48.5 | 127±62.2 | 0.02 |
| IiAUC 0-2h on SAG | -5.58±1.30 | -9.74±5.60 | 0.43 | 163±8.05 | 192±17.3 | 0.25 |
| IiAUC 0-2h on log2(R:S) | 12.5±2.56 | 9.13±6.35 | 0.67 | 98.3±8.03 | 132±28.4 | 0.11 |
| IiAUC 0-3h on RAG | 5.58±1.62 | 1.88±2.35 | 0.37 | -11.5±45.5 | 131±62.5 | 0.02 |
| IiAUC 0-3h on SAG | -5.62±1.21 | -10.4±5.51 | 0.35 | 31.1±7.52 | 205±17.0 | 0.29 |
| IiAUC 0-3h on log2(R:S) | 12.6±2.34 | 9.96±6.26 | 0.74 | 109±7.34 | 139±28.0 | 0.12 |
| HtAUC 0-2h on RAG | -0.72±0.55 | -0.43±0.24 | 0.63 | 141±15.3 | 191±6.45 | <0.001 |
| HtAUC 0-2h on SAG | 0.86±0.45 | 0.74±0.90 | 0.90 | 77.0±2.79 | 70.0±2.78 | 0.01 |
| HtAUC 0-2h on log2(R:S) | -2.28±0.74 | -1.02±0.89 | 0.33 | 88.0±2.31 | 75.9±4.00 | 0.005 |
| HtAUC 0-3h on RAG | -0.74±0.87 | -0.69±0.46 | 0.96 | 160±24.5 | 138±12.2 | <0.001 |
| HtAUC 0-3h on SAG | 1.00±0.75 | 0.83±1.67 | 0.92 | 134±4.63 | 119±5.13 | 0.006 |
| HtAUC 0-3h on log2(R:S) | -2.84±1.35 | -1.34±1.68 | 0.53 | 147±4.24 | 126±7.51 | 0.002 |

GiAUC 0-2h, incremental area under the blood glucose reponse curve over 0-2h; IiAUC 0-2h, incremental area under the serum insulin reponse curve over 0-2h; HtAUC 0-2h, total area under the hunger reponse curve over 0-2h; GiAUC 0-3h, incremental area under the blood glucose reponse curve over 0-3h; IiAUC 0-3h, incremental area under the serum insulin reponse curve over 0-3h; HtAUC 0-3h, total area under the hunger reponse curve over 0-3h. RAG = rapidly available glucose, SAG = slowly available glucose, R:S = RAG:SAG ratio.

P values are for the difference between the Pilot and Granola data.

* Means±SEMs are y-intercepts, p-values are for the elevation or intercept.

### Supplementary Table 4: Regressions of relative glucose/insulin responses on RAG, SAG and log2(RAG:SAG): Pilot vs Granola data.

| Regression | Slope | | | | Elevation or y-intercept* | | |
| --- | --- | --- | --- | --- | --- | --- | --- |
|  | Pilot | Granola | Pooled | P | Pilot | Granola | P |
| RGR on RAG | 1.73±0.70 | 1.34±0.37 | 1.42 | 0.64 | 40.7±19.5 | 56.5±9.9 | 0.11 |
| RGR on SAG | -1.84±0.51 | -4.46±0.56 | - | 0.01 | 98.9±3.1 | 102.1±1.7 | - |
| RGR on log2(R:S) | 4.03±1.11 | 4.71±0.58 | 4.45 | 0.59 | 77.7±3.5 | 72.1±2.6 | 0.12 |
| RIR on RAG | 3.23±0.65 | 0.98±0.30 | - | 0.01 | -7.88±18.3 | 68.3±8.0 | - |
| RIR on SAG | -3.20±0.42 | -3.28±0.52 | -3.22 | 0.91 | 99.7±2.6 | 101.5±1.6 | 0.39 |
| RIR on log2(R:S) | 6.90±1.10 | 3.45±0.54 | - | 0.02 | 63.2±3.4 | 79.5±2.4 | - |
| RHR on RAG | -1.04±0.59 | -0.83±0.21 | -0.88 | 0.72 | 139.1±16.7 | 125.6±5.5 | 0.005 |
| RHR on SAG | 1.17±0.47 | 1.75±1.08 | 1.33 | 0.61 | -89.0±2.9 | -57.0±3.3 | 0.50 |
| RHR on log2(R:S) | -2.94±0.73 | -2.22±0.95 | -2.49 | 0.59 | 118.2±2.3 | 113.1±4.3 | 0.24 |

RGR = relative glucose response; RIR = relative insulin response, RHR = relative hunger response. RGR, RIR and RHR were calculated by expessing the mean incremental area under the curve over 2 h (iAUC02) or, for RHR, the mean total AUC over 3 h (tAUC03) for each test meal as a percentage of that for the test meal with the the maximum mean iAUC02 or tAUC03. RAG = rapidly available glucose, SAG = slowly available glucose, R:S = RAG:SAG ratio.

P values are for the difference between the Pilot and Granola data.

* Means±SEMs are y-intercepts, p-values are for the elevation or intercept.

### Supplementary Table 5: Exploratory multiple regression analysis for Glucose Responses

| Independent Variables | Dependent Variable | | | | |
| --- | --- | --- | --- | --- | --- |
|  | iAUC0-2 | iAUC2-3 | iAUC0-3 | RGR | inc120min |
| Test-meal (Cont=1) | 19.9±5.7^1^  (4.0%)^2^  [16.0±7.9]^3^ | - | 21.2±6.3  (3.7%)  [ns] | 13.1±3.4  (5.7%)  [ns] | - |
| Sex  (M=1) | - | - | - | - | - |
| Ethnicity (Cauc=1) | -14.9±6.6  (2.5%)  [-14.8±6.6] | -6.09±1.48  (5.9%)  [-6.09±1.65] | -23.6±6.9  (3.8%)  [-20.8±7.3] | - | -0.17±0.05  (2.7%)  [-0.15±0.06] |
| Age (y) | 1.24±0.23  (4.5%)  [1.31±0.25] | 0.14±0.06  (2.4%)  [ns] | 1.38±0.25  (4.0%)  [1.43±0.27] | - | 0.0056±0.0021  (2.6%)  [0.0050±0.0023] |
| Height (cm) | -0.94±0.33  (6.0%)  [ns]^5^ | - | -0.91±0.36  (5.6%)  [ns] | - | - |
| Weight (kg) | - | - | - | - | - |
| BMI (kg/m²) | - | - | - | - | - |
| Fasting Glucose (mmol/L) | -34.4±8.6  (5.6%)  [-33.4±8.6] | -6.01±2.15  (1.6%)  [-6.17±2.16] | -41.0±9.6  (6.3%)  [-39.6±9.5] |  | -0.37±0.08  (7.3%)  [-0.38±0.08] |
| Fasting Insulin (pmol/L) | - | - | - | - | - |
| r² for SWMRA^6^  r² for MRA9V^7^ | 0.226  [0.241] | 0.099  [0.126] | 0.235  [0.246] | 0.057  [0.074] | 0.126  [0.137] |

iAUC0-2, iAUC2-3 and iAUC0-3 = incremental area under the curve over 0-2h, 2-3h and 0-3h, respectively (mmol×min/L); RGR = relative glycemic response (iAUC0-2 for Test granola expressed as a % of that for Control granola); inc120min = glucose increment at 2 h (mmol/L); SWMRA = stepwise multiple regression analysis; MRA9V = multiple regression analysis including all 9 variables.

For SWMRA, the order the dependent variables were added to the model is color-coded as shown below. “-“ indicates that the independent variable was not associated (P>0.05) with the dependent variable in either the SWMRA or the MRA9V. df = 239-n, where n= the number of independent variables in the model.

| 1^st^ | 2^nd^ | 3^rd^ | 4^th^ | 5^th^ |
| --- | --- | --- | --- | --- |
|  |  |  |  |  |

^1^ This and similar values: mean±SEM β-coefficient from the SWMRA.

^2^ This and similar values: % of dependent variable variation explained by the independent variable in the SWMRA.

^3^ This and similar values: mean±SEM β-coefficient from the MRA9V.

^4^ “ns” means that the independent variable was not included in the SWMRA but was significant in the MRA9V.

^5^ “[ns]” means that the independent variable was not significant in the MRA9V.

^6^ Proportion of total variation of dependent variable explained by the SWMRA model.

^7^ Proportion of total variation of dependent variable explained by the MRA9V model.

### Supplementary Table 6: Exploratory multiple regression analysis for Insulin Responses

| Independent Variables | Dependent Variable | | | | |
| --- | --- | --- | --- | --- | --- |
|  | iAUC0-2h | iAUC2-3h | iAUC0-3h | RIR | inc120min |
| Test-meal (Cont=1) | 89.7±15.9^1^  (11.5%)^2^  [87.0±16.0]^3^ | - | 94.8±17.4  (7.6%)  [90.8±17.5] | - | 8.02±3.38  (1.6%)  [ns] |
| Sex  (M=1) | -48.2±11.9  (2.1%)  [-46.6±15.4] | - | -52.3±13.1  (3.8%)  [-45.8±16.9] | - | - |
| Ethnicity (Cauc=1) | -43.8±12.2  (3.7%)  [-42.1±13.3] | - | -52.7±13.6  (8.7%)  [-47.9±14.7] | - | -7.23±3.76  (3.2%)  [ns] |
| Age (y) | -1.61±0.48  (2.8%)  [-1.99±0.53] | -0.436±0.088  (4.5%)  [-0.411±0.093] | -1.76±0.53  (4.3%)  [-2.25±0.58] | - | -0.414±0.145  (1.8%)  [-0.436±0.149] |
| Height (cm) | - | - | - | - | -0.495±0.200  (2.6%) [ns] |
| Weight (kg) | - | -0.460±0.137  (3.1%) [ns] | - | - | - |
| BMI (kg/m²) | 12.4±2.32  (4.8%)  [ns] | 3.82±0.65  (5.3%)  [ns] | 14.5±2.55  (7.6%)  [ns] | - | 3.05±0.67  (4.0%)  [ns] |
| Fasting Glucose (mmol/L) | - | 9.99±3.26  (2.7%)  [10.1±3.29] | - | 12.7±6.3  (1.4%)  [15.3±6.8] | 19.4±5.31  (2.2%)  [19.9±5.38] |
| Fasting Insulin (pmol/L) | 3.02±0.44  (7.2%)  [2.94±0.45] | - | 3.04±0.49  (4.3%)  [2.90±0.50] | - | - |
| Analogous Glucose  Response^6^ | 0.746±0.125  (9.8%)  [0.796±0.132] | 0.685±0.094  (16.5%)  [0.640±0.099] | 0.624±0.125  (5.1%)  [0.674±0.131] | 0.604±0.082  (17.9%)  [0.649±0.085] | 31.8±4.20  (18.2%)  [31.2±4.24] |
| r² for SWMRA^7^  r² for MRA10V^8^ | 0.419  [0.428] | 0.321  [0.333] | 0.403  [0.414] | 0.193  [0.228] | 0.329  [0.331] |

iAUC0-2, iAUC2-3 and iAUC0-3 = incremental area under the curve over 0-2h, 2-3h and 0-3h, respectively (pmol×h/L); RIR = relative insulin response (iAUC0-2 for Test granola expressed as a % of that for Control granola); inc120min = insulin increment at 2 h (pmol/L); SWMRA = stepwise multiple regression analysis; MRA10V = multiple regression analysis including all 10 variables.

For SWMRA, the order the dependent variables were added to the model is color-coded as shown below. “-“ indicates that the independent variable was not associated (P>0.05) with the dependent variable in either the SWMRA or the MRA10V. df = 239-n, where n= the number of independent variables in the model.

| 1^st^ | 2^nd^ | 3^rd^ | 4^th^ | 5^th^ | 6^th^ | 7^th^ |
| --- | --- | --- | --- | --- | --- | --- |
|  |  |  |  |  |  |  |

^1^ This and similar values: mean±SEM β-coefficient from the SWMRA.

^2^ This and similar values: % of dependent variable variation explained by the independent variable in the SWMRA.

^3^ This and similar values: mean±SEM β-coefficient from the MRA10V.

^4^ “ns” means that the independent variable was not included in the SWMRA but was significant in the MRA10V.

^5^ “[ns]” means that the independent variable was not significant in the MRA10V.

^6^ For insulin iAUC0-2 = glucose iAUC0-2; for insulin iAUC2-3 = glucose iAUC2-3, etc.

^7^ Proportion of total variation of dependent variable explained by the SWMRA model.

^8^ Proportion of total variation of dependent variable explained by the MRA10V model.

### Supplementary Table 7: Exploratory multiple regression analysis for Hunger responses.

| Independent Variables | Dependent Variable | | | | |
| --- | --- | --- | --- | --- | --- |
|  | tAUC0-2h | tAUC2-3h | tAUC0-3h | inc120min | inc180min |
| Test-meal (Cont=1) | - | - | - | - | - |
| Sex  (M=1) | 17.8±4.71^1^  (6.0%)^2^  [18.0±6.3]^3^ | 8.67±3.02  (3.4%)  [10.5±4.0] | 26.8±7.35  (5.3%)  [29.0±9.76] | 8.20±3.13  (3.0%)  [9.62±4.23] | - |
| Ethnicity (Cauc=1) | - | - | - | -7.04±3.12  (2.0%)  [ns] | -7.26±3.25  (2.1%)  [ns]^5^ |
| Age (y) | - | - | - | - | - |
| Height (cm) | - | - | - | - | - |
| Weight (kg) | - | - | - | - | - |
| BMI (kg/m²) | - | - | - | - | - |
| Fasting Glucose (mmol/L) | ns^4^  [-16.0±7.23] | - | ns  [-24.9±11.3] | - | - |
| Fasting Insulin (pmol/L) | - | ns  [-0.234±0.118] | 0.407±0.203  (1.6%)  [ns] | - | - |
| Analogous Glucose  Response^6^ | -0.093±0.047  (1.5%)  [-0.123±0.053] | - | ns  [-0.155±0.076] | - | - |
| r² for SWMRA^7^  r² for MRA10V^8^ | 0.075  [0.121] | 0.034  [0.089] | 0.069  [0.117] | 0.050  [0.057] | 0.021  [0.043] |

tAUC0-2, tAUC2-3 and tAUC0-3 = total area under the curve over 0-2h, 2-3h and 0-3h, respectively (mm×h/L); inc120min and inc180min = hunger increment at 2 h and 3 h, respectively (mm); SWMRA = stepwise multiple regression analysis; MRA10V = multiple regression analysis including all 10 variables.

For SWMRA, the order the dependent variables were added to the model is color-coded as shown below. “-“ indicates that the independent variable was not associated (P>0.05) with the dependent variable in either the SWMRA or the MRA10V. df = 239-n, where n= the number of independent variables in the model.

| 1^st^ | 2^nd^ |
| --- | --- |
|  |  |

^1^ This and similar values: mean±SEM β-coefficient from the SWMRA.

^2^ This and similar values: % of dependent variable variation explained by the independent variable in the SWMRA.

^3^ This and similar values: mean±SEM β-coefficient from the MRA10V.

^4^ “ns” means that the independent variable was not included in the SWMRA but was significant in the MRA10V.

^5^ “[ns]” means that the independent variable was significant in the SWMRA but not the MRA10V.

^6^ For hunger tAUC0-2 = glucose iAUC0-2; for hunger tAUC2-3 = glucose iAUC2-3, etc.

^7^ Proportion of total variation of dependent variable explained by the SWMRA model.

^8^ Proportion of total variation of dependent variable explained by the MRA10V model.

### Supplementary Figure 3: Associations between relative glucose/insulin responses and RAG, SAG and log2(RAG:SAG): comparison with literature data.

Values are means for test foods from various studies as follows: current pilot (●) and granola (●) studies and Rebello et al, 2020 (^[[3]](#endnote-4)^) (⭘) (red/pink circles were combined since RAG and SAG were measured by PepsiCo and the 3 data points from Rebello fit well with the current data); Peronnet et al. 2015 (^[[4]](#endnote-5)^) (⭘), Vinoy et al. 2013 (^[[5]](#endnote-6)^) (●) and Nazare et al. 2010 (^[[6]](#endnote-7)^) (●) (green circles combined since they involved the same French investigators and fit together well; RAG and SAG were estimated from rapidly digested starch (RDS) and slowly digested starch (SDS) in Perronnet et al.); Garsetti et al. 2005 (^[[7]](#endnote-8)^) (⭘); Englyst 1999 (^[[8]](#endnote-9)^) (⭘); and Englyst 2003 (^[[9]](#endnote-10)^) (●). Anderson et al. 2010 (^[[10]](#endnote-11)^) was not included because the sum of RDS and SDS (theoretically equivalent to available carbohydrate, since the test-meals contained neglible amounts of sugars) in the 4 test meals varied from 14 to 42 grams. Zhu et al. (^[[11]](#endnote-12)^) was not included for several reasons: the sum of RDS and SDS in the 13 different test meals (theoretically equivalent to available carbohydrate, since the test-meals contained neglible amounts of sugars), varied from 16 to 43 grams and was significantly correlated with the glycemic response (r=0.71, n=14, p<0.0001); furthermore, data on RDS and SDS were only shown in stacked bar graphs, making estimation of the amounts imprecise, particularly for the several test meals containing very low amounts of SDS leading, potentially, to very large errors in the estimation of RAG:SAG ratio.

### Supplementary Table 8: Regressions of relative glucose/insulin responses on RAG, SAG and log2(RAG:SAG): comparison with literature data.

| Regression |  | Englyst99  (n=8)* | Englyst03  (n=23) | Garsetti  (n=24) | French  (n=8) | PepsiCo  (n=15) | P** |
| --- | --- | --- | --- | --- | --- | --- | --- |
| RGR on RAG | slope | 1.07±0.55 | 2.12±0.43 | 1.50±0.55 | 0.94±0.30 | 1.62±0.25 | 0.14 |
|  | y-int | 43.1±16.8^ab^ | -11.0±15.4^b^ | 38.3±15.6^a^ | 41.7±13.0^ab^ | 46.5±6.7^a^ | <0.001 |
| RGR on SAG | slope | -1.95±0.49 | -3.53±0.59 | -1.43±0.66 | -1.86±0.15 | -2.35±0.66 | 0.06 |
|  | y-int | 91.7±6.7^ab^ | 85.3±4.1^b^ | 95.6±7.0^ab^ | 101.0±2.0^a^ | 97.8±3.2^a^ | <0.001 |
| RGR on log2(R:S) | slope | 7.92±1.37 | 9.98±1.64 | 7.82±3.35 | 4.35±0.60^1^ | 4.73±0.90^2^ | 0.007 |
|  | y-int | 52.2±5.1 | 34.6±5.5 | 69.1±5.3 | 65.3±2.9 | 73.0±3.4 | - |
| RIR on RAG | slope | - | 1.18±0.30 | 0.73±0.43 | 0.74±0.33 | 1.80±0.50 | 0.17 |
|  | y-int | - | 28.1±10.8^b^ | 67.5±12.2^ab^ | 53.4±14.0^ab^ | 37.0±13.4^ab^ | <0.001 |
| RIR on SAG | slope | - | -1.33±0.53^ab^ | -1.28±0.44^b^ | -1.53±0.33^ab^ | -3.66±0.78^a^ | 0.048 |
|  | y-int | - | 78.1±3.7 | 101.4±4.7 | 100.8±4.6 | 98.2±3.7 | - |
| RIR on log2(R:S) | slope | - | 3.38±1.53 | 6.23±2.34 | 3.55±0.91 | 6.50±1.25 | 0.22 |
|  | y-int | - | 60.1±5.1^b^ | 78.9±3.7^a^ | 71.5±4.4^ab^ | 62.4±4.7^b^ | <0.001 |

RGR = relative glucose response; RIR = relative insulin response. RGR and RIR were calculated by expessing the mean incremental area under the curve over 2 h (iAUC02) or mean glycemic index (GI) for each test meal as a percentage of that for the test meal with the the maximum mean iAUC02 or GI. RAG = rapidly available glucose, SAG = slowly available glucose, R:S = RAG:SAG ratio.

Values are means±SEM of slopes and y-intercepts of the regression lines (calculated using GraphPad Prism version 9.2.1; GraphPad Software, San Diego, CA). Data points and correlations shown in Supplementary Figure 3. Data sources: Englyst99 (8); Englyst03 (9); Garsetti (7); French, combined data from (4,5,6); PepsiCo, combined data from the current Pilot and granola studies and (3).

* number of test meals in the data set.

** P value for heterogeneity of slopes and intercepts (GraphPad Prism).

^ab^ Means not sharing the same letter superscript differ significantly (P<0.05 by t-test).

^1^ Significantly different from Englyst99 (by t-test)

^2^ Significantly different from Englyst03 (by t-test)

### Supplementary Table 9: Multiple regressions of RGR/RIR on log2(RAG:SAG), fat and protein for Englyst03 and Garsetti datasets.

| Study | Dependent Variable | Model^1^ | Independent Variables | | |
| --- | --- | --- | --- | --- | --- |
|  |  |  | Log2(RAG:SAG) | Fat | Protein |
| Englyst03  n=23 foods | RGR | R:S | 9.98±1.64 (P<0.001) | - | - |
|  |  | R:S+F | 7.14±1.90 (P=0.001) | -1.51±0.63 (P=0.03) | - |
|  |  | R:S+P | [10.4±1.72]^2^ | - | (-1.01±1.245)^3^ |
|  |  | R:S+F+P | 7.27±2.13 (P=0.003) | -1.48±0.69 (P=0.045) | (-0.18±1.20) |
|  | RIR | R:S | 3.38±1.53 (P=0.04) | - | - |
|  |  | R:S+F | [3.84±2.01] | (0.25±0.67) | - |
|  |  | R:S+P | [2.55±1.49] | - | (2.20±1.07) |
|  |  | R:S+F+P | [2.18±2.05] | (-0.18±0.66) | (2.30±1.16) |
| Garsetti  n=24 foods | RGR | R:S | 7.80±3.34 (P=0.03) | - | - |
|  |  | R:S+F | [5.46±3.34] | (-1.67±0.82) | - |
|  |  | R:S+P | 7.74±2.99 (P=0.02) | - | -4.73±1.85 (P=0.02) |
|  |  | R:S+F+P | [6.09±3.12] | (-1.18±0.80) | [-3.92±1.88] |
|  | RIR | R:S | 6.23±2.34 (P=0.01) | - | - |
|  |  | R:S+F | [6.74±2.53] | (0.36±0.62) | - |
|  |  | R:S+P | [6.21±2.35] | - | (-1.30±1.45) |
|  |  | R:S+F+P | [7.02±2.53] | (0.57±0.65) | (-1.70±1.52) |

Values are means±SEM of slopes (β-coefficients). RGR = relative glucose response; RIR = relative insulin response. Englyst03 and Garsetti are supplementary references 9 and 7, respectively.

^1^ Independent variables included in the regression analysis: R:S = log2(RAG:SAG); R:S+F = R:S and fat, R:S+P = R:S and protein; R:S+F+P = R:S and fat and protein.

^2^ Means±SEM in () are not statistically significant (P>0.05).

^3^ Means±SEM in [] are not considered valid because all other variables in the model are not significant.

# Supplementary References

1. . Snedecor GW, Cochran WG. Statistical Methods, 7th Edition. Iowa State University Press, Ames, IA, 1980, p 275. [↑](#endnote-ref-2)
2. . Lang DA, Matthews DR, Peto J, Turner RC. Cyclic oscillations of basal plasma glucose and insulin concentrations in human beings. New Eng J Med 1979;301:1023-7. [↑](#endnote-ref-3)
3. . Rebello CJ, Johnson WD, Pan Y, Larrivee S, Zhang D, Nisbet M, Johnson J, Chu YiFang, Greenway FL. A snack formulated with ingredients to slow carbohydrate digestion and absorption reduces the glycemic response in humans: a randomized controlled trial. J Med Food 2020;23:21-28. [↑](#endnote-ref-4)
4. . Péronnet F, Meynier A, Sauvinet V, Normand S, Bourdon E, Mignault D, St-Pierre DH, Laville M, Rabasa-Lhoret R, Vinoy S. Plasma glucose kinetics and response of insulin and GIP following a cereal breakfast in female subjects: effect of starch digestibility. Eur J Clin Nutr 2015;69:740-45. [↑](#endnote-ref-5)
5. . Vinoy S, Normand S, Meynier A, Sothier M, Louche-Pelissier C, Peyrat J, Maitrepierre C, Nazare JA, Brand-Miller J, Laville M. Cereal processing influences postprandial glucose metabolism as well as the GI effect. J Am Col Nur 2013;32:79-91. [↑](#endnote-ref-6)
6. . Nazare JA, de Rougemont A, Normand S, Sauvinet V, Sothier M, Vinoy S, Désage M, Laville M. Effect of postprandial modulation of glucose availability: short- and long-term analysis. Brit J Nutr 2010;103:1461-1470. [↑](#endnote-ref-7)
7. . Garsetti M, Vinoy S, Lang V, Holt S, Loyer A, Brand-Miller JC. The glycemic and insulinemic index of plain sweet biscuits: relationships to in vitro starch digestibility. J Am Coll Nutr 2005;24:441-47. [↑](#endnote-ref-8)
8. . Englyst KN, Englyst HN, Hudson GJ, Cole TJ, Cummings JH. Rapidly available glucose in foods: an in vitro measurement that reflects the glycemic response. Am J Clin Nutr 1999;69:448-54. [↑](#endnote-ref-9)
9. . Englyst KN, Vinoy S, Englyst HN, Lang V. Glycaemic index of cereal products explained by their content of rapidly and slowly available glucose. Brit J Nutr 2003;89:329-40. [↑](#endnote-ref-10)
10. . Anderson GH, Cho CE, Akhavan T, Mollard RC, Luhovyy BL, Finocchiaro ET. Relation between estimates of cornstarch digestibility by the Englyst in vitro method and glycemic response, subjective appetite, and short-term food intake in young men. Am J Clin Nutr 2010;91:932-9. [↑](#endnote-ref-11)
11. . Zhu R, Fan Z, Han Y, Li S, LI G, Wang L, Ye T, Zhao W. Acute effects of three cooked non-cereal starchy foods on postprandial glycemic responses and in vitro carbohydrate digestion in comparison with whole grains: a randomized trial. Nutrients 2019;11:634; doi:10.3390/nu11030634 [↑](#endnote-ref-12)
